# Aptamer-Based Biosensors to Detect Aquatic Phycotoxins and Cyanotoxins

**DOI:** 10.3390/s18072367

**Published:** 2018-07-20

**Authors:** Isabel Cunha, Rita Biltes, MGF Sales, Vitor Vasconcelos

**Affiliations:** 1 CIIMAR/CIMAR—Interdisciplinary Centre of Marine and Environmental Research, University of Porto, Terminal de Cruzeiros do Porto de Leixões, Av. General Norton de Matos, s/n, 4450-238 Matosinhos, Portugal; rita.b.machado@hotmail.com (R.B.); vmvascon@fc.up.pt (V.V.); 2 ISEP—Biomark, Sensors Research, School of Engineering, Polytechnic of Porto, Rua Dr. António Bernardino de Almeida, 431, 4249-015 Porto, Portugal; mgf@isep.ipp.pt; 3 CEB/Centro de Engenharia Biológica, Minho University, Campus Gualtar, 4710-057 Braga, Portugal; 4 FCUP—Department of Biology, Faculty of Sciences, University of Porto, Rua do Campo Alegre, 4169-007 Porto, Portugal

**Keywords:** environmental monitoring, aptasensors, emerging toxins, harmful algal blooms, food and water safety, climate change

## Abstract

Aptasensors have a great potential for environmental monitoring, particularly for real-time on-site detection of aquatic toxins produced by marine and freshwater microorganisms (cyanobacteria, dinoflagellates, and diatoms), with several advantages over other biosensors that are worth considering. Freshwater monitoring is of vital importance for public health, in numerous human activities, and animal welfare, since these toxins may cause fatal intoxications. Similarly, in marine waters, very effective monitoring programs have been put in place in many countries to detect when toxins exceed established regulatory levels and accordingly enforce shellfish harvesting closures. Recent advances in the fields of aptamer selection, nanomaterials and communication technologies, offer a vast array of possibilities to develop new imaginative strategies to create improved, ultrasensitive, reliable and real-time devices, featuring unique characteristics to produce and amplify the signal. So far, not many strategies have been used to detect aquatic toxins, mostly limited to the optic and electrochemical sensors, the majority applied to detect microcystin-LR using a target-induced switching mode. The limits of detection of these aptasensors have been decreasing from the nM to the fM order of magnitude in the past 20 years. Aspects related to sensor components, performance, aptamers sequences, matrices analyzed and future perspectives, are considered and discussed.

## 1. Introduction

Aquatic phycotoxins and cyanotoxins, mostly produced by cyanobacteria, dinoflagellates, and diatoms, can cause an enormous hazard to humans and other animals, contaminating drinking water, water used in agriculture irrigation, for recreational purposes, and to cultivate or simply to sustain the life of aquatic species. Eutrophication, anthropogenic pressure, and global warming are identified factors involved in the increasing occurrence of harmful algal blooms and incidents with emerging toxins. In addition, ballast waters transporting algae spores to new ecosystems or, enormous algae spreading by aquaculture practices, are also involved [1,2,3,4]. Cyanobacteria are responsible for the production of potent toxins, mostly in freshwaters, but with increasing reports in estuaries and coastal waters. These cyanotoxins may have diverse toxic effects being classified as hepatotoxins (microcystins and nodularins), neurotoxins (anatoxin-a, anatoxin-a(s), saxitoxins and analogs and β-methylamino-l-alanine), cytotoxins (cylindrospermopsin) and dermatotoxins (lipo-polysaccharides) [5,6]. In the marine environment, toxins are mostly produced by dinoflagellates, diatoms, and bacteria. They are basically neurotoxic and the most prevalent are regularly monitored in many countries. Among those regulated toxins we should point out okadaic acid, saxitoxins and analogs produced mostly by dinoflagellates, and domoic acid produced by diatoms. Emerging toxins, which mostly occur in tropical regions, are being reported at more temperate waters due to global warming and ballast waters. Examples of emerging toxins are ciguatoxins, brevetoxins, pinnatoxins, spirolides, tetrodotoxin, palytoxin, cyclic imines, among others [7]. These emerging toxins are not yet monitored regularly in most countries because of lack of evidence that they are highly prevalent, and due to technical difficulties for the detection and quantification of some of them [8]. Sensitive, fast and reliable detection and quantification methods are needed. Among these emerging toxins, only brevetoxin-2, palytoxin and tetrodotoxin have been detected by means of aptasensors. 

Aptasensors’ applications in environmental monitoring and food/water safety are an immense area to explore, an alternative to the traditional assays for monitoring of toxins. Because conventional analysis methods have a variety of disadvantages, currently the achievement of simpler, more economical and more efficient alternative methods for toxins detection, has become a stimulating research area. Biosensors are analytical devices used for the detection of an analyte, that include a biorecognition element (bioreceptor), linked with a transducer that transforms the physicochemical information produced into a readout signal, captured by a detector. According to IUPAC in 1999 [9], a biosensor is an integrated receptor-transducer device, which can provide selective quantitative or semi-quantitative analytical information using a biological recognition element. In the case of aptasensors, the bioreceptor is an aptamer. Biosensors have been increasingly used for environmental monitoring [10]. 

Aptamers are single-stranded DNA or RNA oligonucleotides or peptide molecules that bind to a specific target. Oligonucleotides are usually <100 nucleotides long and fold into unique three-dimensional conformations [11,12] of e.g., helices and loops, G-quadruplexes, hairpins, binding to targets with high affinity via van der Waals forces, hydrogen bonding, electrostatic interactions, stacking of flat moieties, and shape complementarities. Contrary to genetic material, characteristics of aptamers are not determined by their nucleotides sequence but by their conformation. Upon target recognition, there is a conformational switching that leads to either an increase or decrease in the signal readout. Despite the type and size of the target, there are other molecular characteristics that determine the specificity by which aptamers can be generated, such as the presence of the target in high concentrations and with high purity. Best candidates include aromatic compounds and hydrogen bond donors and acceptors [13]. Furthermore, the molecule must be resilient to the methodology used to separate target bound/unbound from oligonucleotides during the selection process. 

The systematic evolution of ligand by exponential enrichment (SELEX) is a technology developed for identification and separation of aptamers with specific properties [14], through in vitro screening from random nucleic acid libraries [15]. Aptamers isolated initially by SELEX may not be suitable to use directly, and may follow optimization to improve and regulate their functions [16]. Primary aptamers have been optimized through numerous strategies, e.g., chemical modification, truncation, replacement by locked nucleic acids (LNA), 3′ or 5′ capping and mutation [16,17,18,19,20]. Second generation analogs of aptamers are increasingly being used for molecular targeting, such as X-Aptamers, SOMAmers, LNAs, and others. In the case of SELEX, the chemical diversity is limited to the four DNA and RNA bases while in X-Aptamers technology, the diversity available for target interaction is exponentially expanded, achieving greater specificity and affinity. X-Aptamers are developed using a patented (AM Biotechnologies LLC, University of Texas System) microbead-based selection process in libraries, that provides a combinatorial chemistry platform of 10^8^ unique sets of molecules or more [21]. X-Aptamers are superior targeting agents as they provide an unlimited number of functional groups [22]. Up to our knowledge, X-aptamers have not been used to target aquatic toxins so far.

Due to the intrinsic low stability, RNA aptamers are less suitable for environmental monitoring applications, although their stability may be greatly increased by covalent binding to a support through π-π staking, as e.g., to graphene oxide (GO) nanosheets [23]. Two paths of GO protecting RNA from nucleases are forwarded, the steric hindrance that could hinder the access of nucleases to RNA, due to the covalent immobilization or/and non-covalent π-π stacking between RNA and GO, and GO that may directly inactivate nucleases and then protect RNA [23]. The use of aptamers as recognition elements on sensors for toxin detection has advantages when compared to other bioreceptors. Favorable characteristics and advantages that aptamers offer over other biorecognition elements and toxin detection methodologies are summarized in Table 1.

Toxins may kill the organism or cells used to produce antibodies, while no toxic effects are possible on aptamer synthesis. Real-time aptasensors seem to be an ideal tool for aquatic toxins monitoring, as results are provided in a more immediate way, which suits best the management of urgency and risk that those toxins pose to human, animal or ecosystems health. They also suit risk assessment associated with long-term exposure to low concentrations of toxins, since their limits of detection tend to be as low as (or lower than) conventional laboratory methods performed with heavy equipment and highly skilled staff and, below the detection limits imposed by the legislation. They may be mounted on high throughput multiplexed sensitive detection platforms for routine analysis of water samples, in the field, e.g., in oceanographic buoys, due to their stability at ambient temperature and minimal sample preparation needed. 

The transduction of the binding event between the aptamer and the target molecule, into a measurable signal, is equally important to produce a biosensor as the ability for an aptamer to bind the analyte [24]. Biosensors are normally classified according to the mode of signal transduction employed. The transduction may be electrochemical, optical, mass-based or calorimetric [25,26]. Only electrochemical and optical aptasensors have been described in the literature to have been used so far to detect aquatic toxins.

*Electrochemical biosensors* measure the electrical properties of a solution, produced in a biochemical reaction, between biomolecules and target analyte that yield or consume ions or electrons [27]. Biochemical sensors usually contain two components connected in series: a biochemical recognition element, herein aptamers, and a physicochemical transducer. They can be amperometric, potentiometric, impedimetric and ion-change or field effect. The working electrode(s) are usually made of gold [28,29,30,31], indium tin oxide [32], or carbon-based compounds [23,33,34,35], screen-printed or with a self-assembled layer on top [36,37,38], further covered by the bioreceptor. Amperometric sensors measure the production of a current when a potential is applied between two electrodes. The current is typically from nA to μA range. Potentiometric sensors measure the difference in potential (voltage) between two electrodes, a working electrode and reference electrode, generated by two solutions, separated by an ion-selective membrane, at virtually zero current flow. The reference electrode is needed to provide a defined reference potential. Conductometric (impedimetric) biosensors measure changing electrical conductance/resistance of a solution due to ions or electrons produced during a biochemical/chemical reaction. Ion-charge or field effect biosensors use an ion-sensitive field-effect transistor (ISFET) to determine ion concentrations. An ISFET is composed of an ion-selective membrane where to the biorecognition element is coupled, and allowing specific ions to pass through for detection. 

*Optical biosensors* may measure absorption, fluorescence, luminescence, internal reflection, surface plasmon resonance (SPR), or light scattering spectroscopy. Optical biosensing can be broadly divided into two general modes: label-free and label-based [39,40]. In the label-free mode, the detected signal is generated by the interaction of the analyte with the transducer, exploiting changes that occur in the optical property of the biomolecule [41], such as absorbance, emission, polarization, or luminescence decay time of a receptor. Also, SPR, evanescent wave fluorescence, and optical waveguide interferometry detect the interaction of the probe with the target without any label, using the evanescent field close to the biosensor surface. The label-based mode makes use of optical labels and probes of various kinds to generate a colorimetric, fluorescent or luminescent optical signal, enabling the shift of the analytical wavelength(s) to nearly any value [39]. Colorimetric (absorption) sensors are a good alternative for rapid portable measurements in the field for environmental monitoring or food quality inspection, because these sensors may be detected with a simple portable spectrometer, with a naked eye, or with image analysis applications installed on a smartphone upon photography acquisition [42,43,44,45,46], in the two latter, only in wavelengths of the visible light.

Biosensors have also been classified according to their design strategy [47] into target-induced structure switching mode, sandwich or sandwich-like mode, target-induced dissociation/ displacement mode and competitive replacement mode:

1. In target-induced structure switching mode (TISS), the target binds directly to their aptamers, inducing a conformational switch of aptamers, leading to changes on a detectable signal such as weight, fluorescence, color, redox state, conductivity, among others. Most of the sensors analyzed in the present review, and so far developed to detect aquatic toxins, are of this mode [23,28,29,30,31,32,33,34,35,36,38,48,49,50,51,52,53,54,55,56,57].
2. Sandwich or sandwich-like sensors mode (SS) takes advantage of the fact that some targets, have two binding sites, which allows them to bind to two recognition elements and form sandwich-like complexes. In the case of aptasensors, this mode can have two formats: aptamer-protein-aptamer and aptamer-protein-antibody. No sensor of this mode was found to have been used to detect aquatic toxins.
3. Target-induced dissociation/displacement (TID) mode uses complementary sequences of aptamers. After incubation with target molecules, the aptamer binds to the target and is liberated from its complementary sequence. Four TID sensors were found concerning aquatic toxins detection [58,59,60].
4. Competitive replacement mode (CR) relies on the replacement of signal modified analytes bound to surface-bound aptamers by the analyte in solution. Signal modified analytes need to be designed and synthesized. After incubation with the non-signal modified target analyte (sample), the signal modified molecules are released from the aptamer into solution, giving rise to a signal related to the target molecule concentration. Another form of this competitive mode consists in replacing the surface bound analyte by the analyte in solution, to modified aptamers that emit or quench a signal [37,60,61,62].

Aptamer immobilization plays a key role in the performance of the sensor. Various immobilization techniques for biorecognition elements are defined and classified by Labuda et al. [63] and Korotkaya et al. [26] in categories such as physical or chemical adsorption, microencapsulation, inclusion, cross-linking and affinity binding. During the aptamer selection process, when the analyte is bound to a surface to bind the aptamers’ library, or even in some aptasensors where its architecture implies that the analyte is bound to e.g., transducer, electrode, the immobilization process of the molecule need to allow for exposure of the molecular zone targeted by the aptamer. Moreover, in molecules with various congeners, e.g., microcystins, the selected aptamer needs to target the variable regions to feature adequate selectivity, and immobilization mode chosen needs to allow for exposure of the variable regions [28].

Nanomaterials such as single-walled carbon nanotubes, titanium dioxide nanotubes, graphene oxide nanosheets, molybdenum disulfide nanosheets, graphene hydrogels, gold nanoparticles, upconversion lanthanide nanoparticles, quantum dots, among others, have been used to improve the sensitivity of biosensors, by amplifying the corresponding signals. Low concentration safety values imposed by national and international health organizations/agencies, as well as, the new challenges posed by climate change, call for simple, rapid, sensitive and reliable detection methods, that aptasensors can deliver, contributing to achieve UN 2030 AGENDA goals, namely on what concerns clean water availability for all and sustainable use of water and safe food for all. The different architectures that have been used in aptasensors developed to detect aquatic toxins are presented and analyzed in the next section.

## 2. Aptasensors for Aquatic Phycotoxins and Cyanotoxins Detection

The type of aptasensor, the design strategy, the materials used, the aptamer immobilization methods, and the performance characteristics of the various aptasensors that have been developed so far for detection of aquatic phycotoxins and cyanotoxins are described at continuation and summarized in Table 2. The characteristics of the aptamers selected, identifying modifications at 3′ or 5′ ends to label or link the aptamers to the sensor platform, and their affinity to the target toxin are summarized in Table 3.

### 2.1. Freshwater Toxins

#### 2.1.1. Anatoxin-A (ATX) 

Anatoxin-a (ATX-A) is a neurotoxic alkaloid secondary amine produced by some strains of freshwater cyanobacteria of the genera *Anabaena*, *Arthrospira*, *Aphanizomenon*, *Cylindrospermopsis*, *Oscillatoria*, *Phormidium*, and *Raphidiopsis*. ATX-A and homoanatoxin-a (HATX) are the most commonly occurring anatoxins, with a molecular weight of 165.232 and 293.282 g/mol, respectively. These are strong nicotinic acetylcholine receptor antagonists mimicking acetylcholine, binding to acetylcholine receptor from where the cell machinery is unable to remove it, bringing nerve and muscles to exhaustion. In high doses, they may eventually result in respiratory muscles paralysis and death [64].

**Figure 1:**
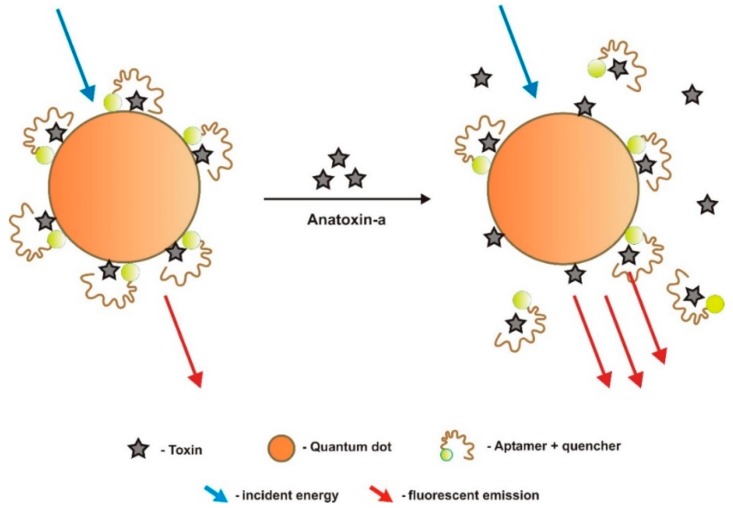
Schematic of the design strategy for the optical fluorescent resonance energy transfer (FRET) competitive binding assay developed by Jackson et al. [61], to detect anatoxin-a (ATX-A), based on quantum dot nanocrystals with ATX-A bound on their surface, and aptamers to ATX-A conjugated with quenchers of QDs fluorescence.

Jackson et al. [61] developed a sensor to detect ATX-A based on responsive fluorescent resonance energy transfer (FRET). The feasibility of this optical sensor was tested with thyroxin, a similar size molecule, but not really with ATX-A, due to its high toxicity. Thus, it cannot be considered an ATX-A biosensor. The aptamer sequence was not disclosed, and the sensor performance data were not shown. Aptamers conjugated to terminal quencher fluorophore dyes are the molecular recognition elements, in a competitive-binding assay (Figure 1). ATX-A molecules were immobilized on the surface of highly stable and fluorescent quantum dot (QD) semiconductor nanocrystal nanoshells (~2–100 nm) by covalent bonds, covered by a polyethylene glycol (PEG) coating. Once bound to the ATX-A immobilized on the QD surface, aptamers quenched QDs fluorescence. Upon exposure of this reagent mixture to free ATX, the quenching aptamers are released from the QDs surface and the fluorescence is restored, producing a signal proportional to the ATX concentration in the measured sample. The primary amine of thyroxin (T4) was covalently immobilized on the carboxyl terminated QDs that fluoresce at 655 nm (QD655, Invitrogen, Carlsbad, CA, USA) using 1-ethyl-3-(3-dimethylaminopropyl) carbodiimide hydrochloride/*N*-hydroxysuccinimide (EDC/NHS) chemistry. A carboxyl version of the quenching chromophore QSY21 (Invitrogen) was conjugated to the DNA aptamers synthesized with a 5′-amine termination. A Qubit™ portable fluorometer (Invitrogen) was used to acquire data, a system that suits data collection in the field or remote low-tech laboratories. Authors highlighted significant advantages provided by QDs over organic fluorophores, such as size-tunable photoluminescence spectra, higher quantum yields, broad absorption, narrow emission wavelengths [70,71], and the much-increased resistance of QDs to photobleaching, that allows more reproducible and stable assays.

**Figure 2:**
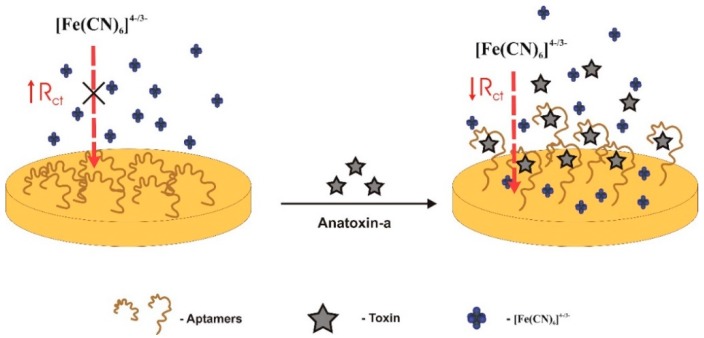
The mode of action of this impedance aptasensor is based on the decrease of electron resistance when aptamers change from a random conformation to a compact conformation in the presence of anatoxin-a [31]. R_ct_: charge-transfer resistance.

To produce an impedance aptasensor for ATX-A detection, Elshafey et al. [31] used a monolayer of aptamers self-assembled on a gold electrode (Figure 2). Aptamers were disulfide modified [HO−(CH_2_)_6_S−S−(CH_2_)_6_−DNA]). The self-assembled monolayer (SAM) was modified by mercaptohexanol (a short alkanethiol) to displace those aptamers weakly adsorbed from the gold surface, and backfill, leaving only aptamers that were covalently bound via their thiol moieties. Aptamer binding was monitored through the [Fe(CN)_6_]^4−/3−^ standard redox probe. In the absence of ATX-A, the negatively-charged redox probe was repelled from the electrode’s surface and its redox reaction is hampered, thereby increasing the resistance to charge-transfer on the gold support. Upon ATX-A recognition, aptamers switched into more compact structures, enabling the contact of the redox probe to the surface, thereby decreasing the resistance to electron transfer, which was used as the sensor signal. The sensor showed a LOD of 0.5 nM, below the guideline value of 1 μg/L for water safety [72]. The linear range of detection (LRD) was between 1 nM and 100 nM, with a dissociation constant, K_d_, of 27.14 ± 75.38 nM. 

#### 2.1.2. Cylindrospermopsin (CYN)

Cylindrospermopsin (CYN) is a polycyclic uracil derivative alkaloid produced by some strains of freshwater cyanobacteria of the genera *Anabaena*, *Aphanizomenon*, *Cylindrospermopsis*, *Lyngbya*, *Raphidiopsis*, and *Umezakia*. CYN, deoxycylindrospermopsin (deoxyCYN), and 7-epicylindro-spermopsin (epiCYN) are the known occurring CYN variants, with a molecular weight of 415.421, 399.420 and 415.421 g/mol, respectively. The loss of the uracil group from CYN removes its toxicity. It is zwitterionic, being highly water-soluble. CYN is cytotoxic, genotoxic, dermatoxic, and tumor promoter. Its mode of action is through the inhibition of protein synthesis and covalent modification of DNA and/or RNA, being mostly toxic to the liver but also affecting kidney, lungs, intestine, stomach, and circulatory system.

The first aptasensor targeting CYN was a label-free impedimetric aptasensor developed by Elshafey et al. [30]. This electrochemical sensor is similar in architecture to that described above to target ATX-A, and developed by the same research group [31]. Upon CYN being captured by the aptamer, there is a reduction of the electron transfer resistance measured. The sensor showed a LOD of 0.039 μg/L, which is well below the safety guideline for CYN in drinking water, which is 1 μg/L in some countries (2.564 nM) [73]. The LRD ranges between 0.1 nM and 80 nM and the aptamer binding featured a K_d_ of 20.25 ± 2.7 nM.

Another label-free impedance-based aptasensor targeting CYN was developed by Zhao et al. [33], but at this time using thionine–graphene (TH–G) nanocomposites on the electrode. The aptamer was amino-substituted at one end and covalently attached to the surface of the TH–G nanocomposite modified glassy carbon electrode (GCE) through the cross-linker glutaraldehyde (GA), yielding a sensing surface of aptamer/GA/TH–G/GCE. The sensor showed a LOD of 300 pM (0.117 μg/L) and a LRD between 1.0 nM and 200 nM (0.39 and 78 μg/L). CYN induced a change in conformation of the aptamer, increasing the accessibility of the redox couple to the electrode surface, thereby decreasing charge transfer resistance. This aptasensor had a good reusability and stability. In the presence of 1 nM okadaic acid and MC-LR, it showed good selectivity.

#### 2.1.3. Microcystins

Microcystins (MCs) are monocyclic heptapeptides consisting of five constant amino acids and two variable ones e.g., MC-LR contains leucine (L) and arginine (R) at the two variable positions, and a MW of 995.171 g/mol. Four MCs (MC-LR, -RR, -LA and -YR) are on US Environmental Protection Agency (US EPA) Contaminant Candidate List III [74] and are of special concern to the US EPA. MCs are mostly produced by cyanobacteria of the genera *Microcystis*, *Anabaena*, *Phormidium*, *Nostoc*, and *Planktothrix*. There are more than two hundred known MC congeners [75] which exhibit structure variations and differ in toxicity, being the most prevalent MC-LR, also the first MC chemically identified. Apart from varying in the amino acids of the two variable positions, MCs differ on the degree of methylation, hydroxylation, epimerization, and consequently, on toxicity [76,77]. MCs are hepatotoxic upon drinking contaminated water and foodstuffs, or skin contact, being also a potential tumor promoter and carcinogen. MCs inhibit protein phosphatase 1A and 2A (two key enzymes in cellular processes) to varying degrees, causing high levels of protein phosphorylation and cytoskeletal collapse. The World Health Organization (WHO) guidelines indicate 1 μg/L in drinking water and a Tolerable Daily Intake (TDI) of 0.04 μg/kg body weight per day for MC-LR [78,79]. MC-LR levels lower than 1 μg/L can significantly interrupt cellular processes [80]. These regulations and data call for simple, rapid, sensitive and reliable MC detection methods, suitable for field use.

Nakamura et al. [48], in 2001, selected the first aptamers targeting MC-LR. It was used in an optical aptasensor based on surface plasmon resonance (SPR). Biotin-modified aptamers were attached on a sensor chip (Biacore International AB, Uppsala, Sweden) through affinity of biotin-avidin, and an SPR apparatus, BIACORE X (Biacore International AB), was used to detect the MC-LR. Despite the target molecule being MC-LR, the aptamer had higher specificity to MC-YR (Y-tyrosine; R-arginine) than to MC-LR. Authors concluded that the aromatic ring of tyrosine (YR) was more responsible for binding than the leucine’s (LR) alkyl chain. The detection range was 50 to 1000 mg/L and the affinity was low, with K_d_ = 10^3^ M^−1^. The parameters obtained were lower than those of other methodologies in use at that time, namely those using antibodies and protein phosphatases; however, this work was an early demonstration of the potential use of aptamers on toxin detection.

Quite later, in 2012, Ng et al. [28] reported the development of novel MC-targeting aptamers. One aptamer displayed high affinity and specificity to MC-LR, -YR, and -LA, simultaneously, with K_d_ ranging from 28 to 60 nM. Two other aptamers were selected, one specific to MC-LR, and another simultaneously specific to MC-LA and -LR. These selectivity results implied that the selected aptamers were targeting variable regions from the MCs. Authors underline the importance of letting the variable regions of the target molecules exposed after immobilization to increase selectivity. The three aptasensors, each constructed with a different aptamer, showed differences in response for different MC analogs on a voltammetric electrochemical platform. Aptamers were self-assembled on a gold electrode via thiol chemistry and exposed to the redox marker cations [Ru-(NH_3_)_6_]^3+^. The cations bind electrostatically to the negatively charged phosphate backbone of the DNA of the aptamer modified surface, resulting in a reduction on the peak current in the square wave voltammetry (SWV) measurements. The decrease in current is possibly due to a change of the aptamers conformation, resulting in less [Ru-(NH_3_)_6_]^3+^ bound to the electrode. The LRD of the aptasensors ranged from 7.5 to 12.8 pM, below the WHO (1998) drinking water maximal concentration of 1 μg/L for MC-LR, indicating the potential use of this aptasensor for water analysis. LRD was between 0.01 nM and 10 nM for the 3 aptamers.

Hu et al. [23] characterized the adsorption capacity and selectivity of covalently immobilized RNA aptamers [81] on graphene oxide (GO), to procedure polydisperse and stable RNA–graphene oxide (RNA-GO) nanosheets that specifically recognized and adsorbed trace MC-LR in drinking water. The nanosheets had a large surface area (2630 m^2^ g^−1^) and very low toxicity, important features to water purification. The maximum adsorption capacity of RNA–GO was 1.44 mg/g, decreasing at extreme pH, temperature, ionic strength, and in the presence of natural organic matter. A specificity experiment using MC-LR, MC-RR, MC-LW, and nodularin, showed that more than 95% of MC-LR was absorbed by the smart RNA–GO and, less than 12% of the other toxins. Regeneration of the MC-LR loaded RNA–GO was achieved in hot pure water at 50 °C for 10 min. The adsorption capacity was reduced after five regeneration cycles by less than 10%. No sensor was developed by these authors; however, the potential of RNA-GO to purify water, concentrate toxins in biological or environmental samples and, possibly as recognition element in biosensors was shown. Covalent immobilization of RNA on GO nanosheets increases its stability. Authors proposed two possible mechanisms for this increased stability: (1) the steric hindrance could protect RNA from nucleases owing the covalent immobilization and the noncovalent π-π stacking; and (2) GO could inactivate nucleases. They concluded that the interactions of RNA–GO with RNase needed further studying.

Lin et al. [29] have also developed an electrochemical impedance aptasensor for MC-LR detection, by immobilizing covalently the aptamers on the gold electrode through Au–S interaction. Binding of the toxin to aptamers resulted in impedance decreasing that presented a linear trend with the logarithm of the MC-LR concentration, in the range of 0.05–100 nM. LOD was 0.018 nM. The sensor showed good selectivity, tested against the lower concentration of MC-RR. High concentration of MC-RR could cause interference; however, little MC-RR interference is expected in real samples since MC-RR concentration tends to be far lower than MC-LR. 

Eissa et al. [36] designed a very sensitive and selective aptasensor for MC-LR detection which utilizes label-free DNA aptamers noncovalently assembled on graphene-modified screen-printed carbon electrodes (SPEs). The DNA aptamers assemble on the electrode giving rise to a marked reduction in the square wave voltammetric signal of the [Fe(CN)_6_]^4−/3−^ redox couple. In the presence of MC-LR, there was a dose-responsive rise in peak current, allowing the quantification of MC-LR. The LOD for this method was 1.9 pM. Excellent selectivity was observed when the sensor was tested against okadaic acid (OA), MC-LA and -YR, despite the aromatic rings in OA and MCs structures that could be adsorbed by π-π stacking on the graphene surface. It was demonstrated that part of the aptamer had changed conformation after coupling to the toxin, without the complete release of the bound pair from the graphene surface. Authors highlighted that the eventual release of the aptamer from graphene surface upon binding would not lead to universal detection architectures of small target molecules, such as some aquatic toxins, which are less likely to cause a change in the entire aptamer’s sequence conformation, and cause the release of the bound unit from the graphene surface. The stability of the aptamer-graphene electrodes was studied over a period of 2 weeks and no loss of signal was observed. A 2.9% decrease in response was observed after one month.

**Figure 3:**
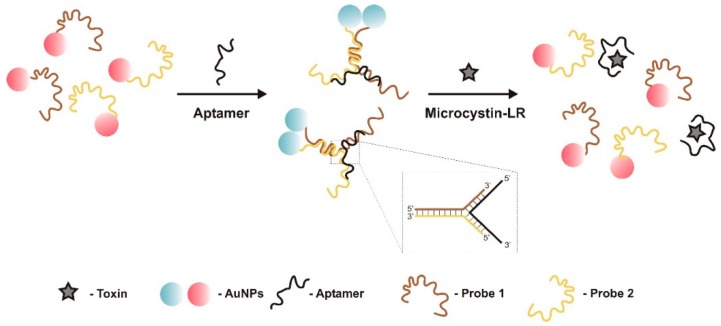
In the presence of the aptamer, the two DNA probes and the aptamer form a Y-shaped DNA duplex, which maintains DNA probes linked and gold nanoparticles (AuNPs) near, forming a dimer [58]. When microcystin-LR is added, the aptamers leave the DNA probes and link to the toxin, changing the structure. The pre-formed AuNP dimers disassemble to monomers and, solution color changing from blue to red.

A colorimetric sensor for detection of MC-LR was developed by Wang et al. [58] using specific aptamer sequences as linkers to prepare gold nanoparticle (AuNP) dimers (Figure 3). AuNPs have oligonucleotides immobilized on their surface, which have complementary sequences to a part of the linker aptamer. The two DNA probes and the linker aptamer form a Y-shaped DNA duplex in the presence of the aptamer, which keeps the DNA probes linked and the AuNPs adjacent. Then, in the presence of MC-LR, the aptamers change structure causing the pre-formed AuNP dimers to disassemble into monomers and, solution color changing from blue to red. Absorbance measurements were conducted in a portable spectrometer. Nearly 5% of AuNPs surface was modified with DNA probe 1 or probe 2, and ~95% with PEG. This asymmetrical modification was to preclude the formation of large AuNP aggregates after DNA aptamers addition. Controlled formation of AuNP dimers, rather than aggregates, greatly benefited the stability of this type of sensors, which have an overwhelming sensitivity and stability. The LOD was 0.05 nM and the LRD between 0.1 nM and 250 nM. Sensitivity was investigated using MC-LA and -YR, and only MC-LR could cause a significant change in absorbance.

**Figure 4:**
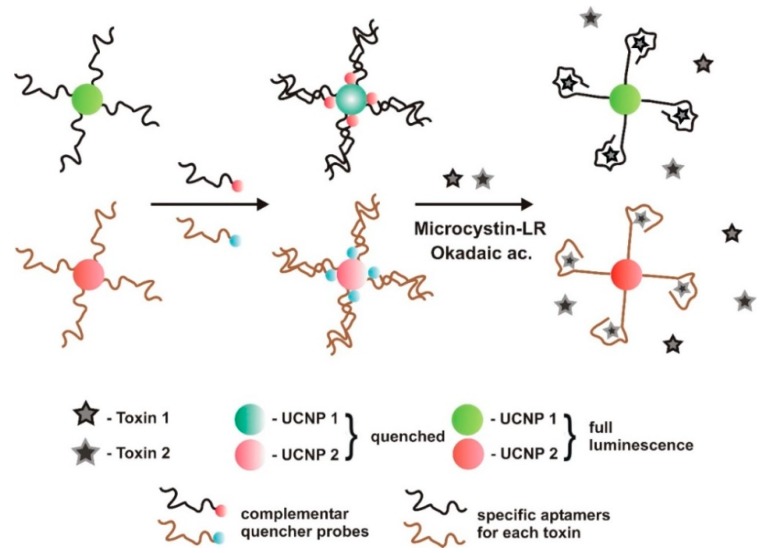
Dual fluorescent resonance energy transfer (FRET) aptasensor, developed by Wu et al. [59] to monitor microcystin-LR and okadaic acid, simultaneously. In the presence of these toxins, the aptamers prefer to bind to them and de-hybridize from the complementary DNA, preventing green and red luminescence quenching. UCNP—Upconversion nanoparticle.

A dual FRET aptasensor was developed by Wu et al. [59] to monitor simultaneously MC-LR and OA, employing green and red upconversion nanoparticles (UCNP) luminescence as the donors, and two quenchers as the acceptors (Figure 4). Lanthanide-doped near-infrared (NIR)-to-visible UCNPs can emit strong visible luminescence with the excitation light typically at 980 nm [82]. Biotinylated aptamers against each toxin were conjugated to different UCNPs. NaYF4: Yb, Ho UCNPs were linked with aptamers against MC-LR and NaYF4: Yb, Er/Mn UCNPs with aptamers against OA. UCNPs have many advantages over other organic fluorophores due to their optical and chemical features, such as tunability, the absence of auto-fluorescence, and the light-scattering background that can be induced by samples. Multicolor UCNP can be used to build multiplex biosensors for the detection of various analytes simultaneously [83]. The two donor-acceptor pairs were produced by hybridizing the aptamers with their complementary DNA. The upconversion luminescence was quenched by quenchers (BHQ1 and BHQ3 from SIGMA, St. Louis, MO, USA) of high overlapping spectrum, attached to oligonucleotide probes (AATGAGGTGGTATGGGTAATTGTCATGGTGGTCCTGTTTG-BHQ3 for MC-LR and TCGTCACATTGACCCTTCGCGTAGCGCTCCCTGTTGTTGG-BHQ1 for OA). When analytes are added, the corresponding aptamers de-hybridize from the complementary DNA to bind to them, preventing the quenching of green and red luminescence. The relative luminescence intensity increased proportionally with increasing toxins concentration, allowing their quantification. The LODs for MC-LR and OA were 0.025 and 0.05 μg/L, respectively. The LRD concentration ranged from 0.1 to 50 μg/L for both toxins. Good recovery and repeatability rates (RSD) were found, 6.47% for MC-LR and 6.24% for OA. This dual FRET assay is robust to interference and has excellent specificity, as tested with DTX-1 and DTX-2 for OA aptamer, and MC-LA and MC-YR for MR-LR aptamer.

Du et al. [32] conceived an aptasensor for quantitative MC-LR detection based on bismuth oxybromide nanoflakes (BiOBr-NF) and nitrogen co-doped graphene (NG), as a photoelectrochemical (PEC) transducer platform, in which the aptamers were immobilized on an indium tin oxide electrode (ITO) support. BiOBr (photocatalyst) promotes the photocatalytic degradation of MC-LR [58,84,85]. In the presence of MC-LR, the aptamers immobilized on the BiOBrNFs-NG ITO electrode capture the toxin on the surface of the sensor, increasing the photocurrent response proportionally to its concentration. The observed photocurrent increase was attributed to a greater amount of MC-LR molecules captured and involved the PEC process, which were quickly oxidized by the photoinduced holes of BiOBr. The current was amplified by the retarded recombination of photoinduced electrons and holes [32]. The LRD of the sensor was from 0.1 pM to 100 nM, with a LOD of 0.03 pM. Specificity was studied against MC-LA and MC-YR, being obtained significant photocurrent changes mostly in the presence of MC-LR. Recovery of MC-LR in fish spiked matrices ranged from 97.8 to 101.6%, with RSD of 2.52–5.14%.

**Figure 5:**
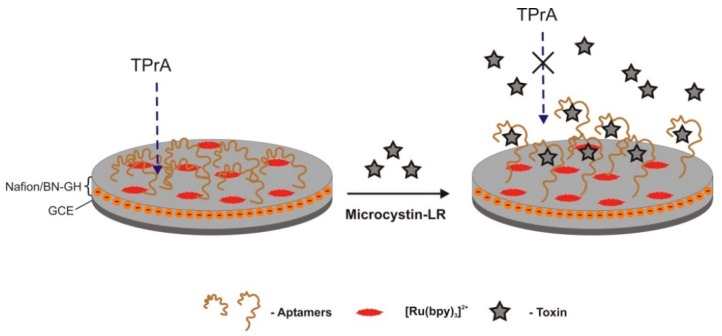
This optical aptasensor is a “signal-off” sensor developed to detect microcystin-LR [34]. When microcystin-LR is incubated with aptamers it blocked the approaching of the coreactant tripropylamine (TPrA) to *tris*(2,2′-bipyridine) ruthenium (2+) ion (Ru(bpy)_3_^2+^) of the glassy carbon electrode (GCE) interface that results in an intense decrease in electrochemiluminescence emission.

The same research group [34], has changed the detection methodology of the aptasensor developed previously [32], to validate the effect of the aptamer-MC-LR assembled to a quartz crystal microbalance (QCM); the 2D graphene layer was replaced by a three-dimensional (3D) graphene hydrogel, co-doped with boron and nitrogen (BN-GH), self-assembled on a *tris*(2,2′-bipyridine)ruthenium (II)-luminophore immobilization platform, to increase aptamers loading due to the nanoporous structure and large specific surface area of the new sensor. Three-D graphene hydrogels (GHs) can supply multidimensional electron transport pathways and be very suitable to assemble more electrochemiluminescent (ECL) molecules on its surface for enhancing its ECL intensity [86,87]. The sensor is a “signal-off” switch system to the Ru(bpy)_3_^2+^−TPrA (tripropylamide) (Figure 5). When MC-LR was incubated with the aptamers, the ECL signals detected by an electrochemiluminescence analyzer decayed intensely as the toxin/aptamer couples blocked the approaching of the coreactant TPrA to the Ru(bpy)_3_^2+^ on the electrode interface. This novel ECL biosensor displayed high sensitivity and selectivity, with a LOD of 0.03 pM and a LRD ranging from 0.1 to 1000 pM.

**Figure 6:**
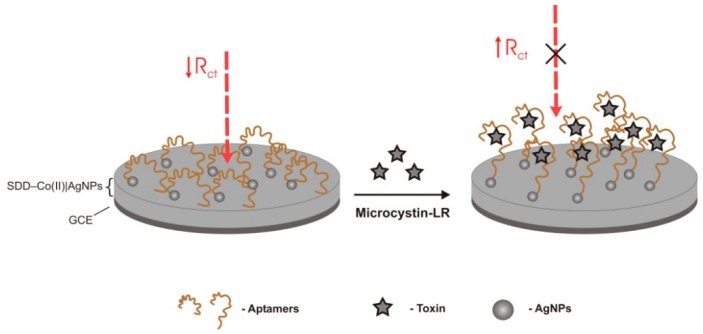
Electrochemical aptasensor for microcystin-LR (MC-LR) detection using a glassy carbon electrode (GCE) with aptamers self-assembled on a surface of cobalt (II) salicylaldimine metallodendrimer (SDD–Co(II)) doped with silver nanoparticles (AgNPs), by their 5′ thiolated end [38]. When aptamer-MC-LR complexes are formed, there is a decrease in the peak current. R_ct_—charge transfer resistance.

Another electrochemical aptasensor to target MC-LR was built by Bilibana et al. [38] research group, using a GCE with a surface of cobalt (II) salicylaldimine metallodendrimer (SDD–Co(II)) doped with silver nanoparticles (AgNPs), where 5′-thiolated DNA aptamers self-assembled (Figure 6). Dendrimers are a class of polymers consisting of a core, self-replicating branching units, and peripheral surface groups [88]. Dendrimers can be entirely modified with functional groups at different positions. In the case of metallodendrimers, the structure is connected to transition metal complexes. The redox reactions of the metal complex and the organic dendrimeric structure organize a molecular electronic communication, on a conductive platform that supports the transport of electrons and increases the sensitivity of the biosensor [89,90,91]. AgNPs have a large specific surface area and the capacity to transfer photoinduced electrons at the surfaces of colloidal particles rapidly, assisting on direct electron transfer [92,93]. The decrease in the peak current indicates aptamer-MC-LR complexes formation. The aptasensor showed a linear response for MC-LR detection between 0.1 μg/L and 1.1 μg/L and the LOD was 0.04 μg/L. The great sensitivity of the sensor was attributed to the AuNPs and to the nanocomposite on the surface of the GCE, which significantly increased aptamers loading. The analysis of spiked samples showed average recoveries that ranged from 94 to 115% with RSD less than 5% (*n* = 3). The aptasensor was very efficient in distinguishing between MC-LR and other toxins, and between MC-LR and MC congeners. Therefore, the alkyl chain of MC-LR’s leucine seems to play a greater role on affinity than the heteroatom chain of MC-RR and the aromatic ring of tyrosine of MC-YR [38].

**Figure 7:**
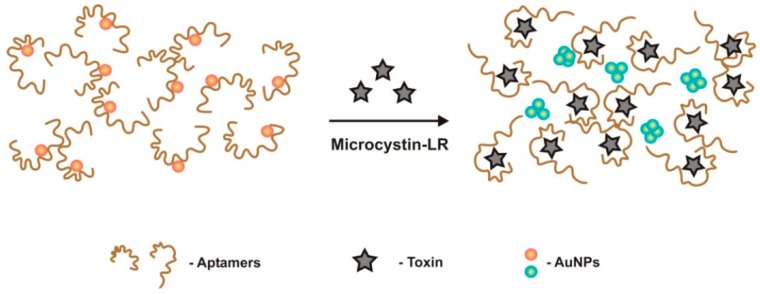
Highly sensitive colorimetric aptasensor for detection of microcystin-LR (MC-LR) [49]. Gold nanoparticles (AuNPs) plasma resonance absorption red peak shifts upon binding of the target. Since the random coil aptamer adsorbed on AuNPs change into the regulated structure, forming MC-LR-aptamer complexes, which cause the release AuNPs from the surface of aptamers, leading to their aggregation, and the color shift from red to blue.

A simple and highly sensitive colorimetric aptasensor was developed by Li et al. [49] for the selective detection of MC-LR. In this, the plasma resonance absorption of AuNPs red peak shifts to blue upon binding of aptamers to MC-LR, at a high concentration of NaCl (Figure 7). The random coiling of aptamers alters into a regulated structure, to form MC-LR-aptamer complexes that release AuNPs, leading to NPs aggregation and subsequent color change. This sensor exhibited a LRD ranging from 0.5 nM to 7.5 μM, and a LOD of 0.37 nM, presenting a highly selective performance in the presence of interfering substances (acetaminprid, glyphosate, dylox, atrazine, and clofentezine).

**Figure 8:**
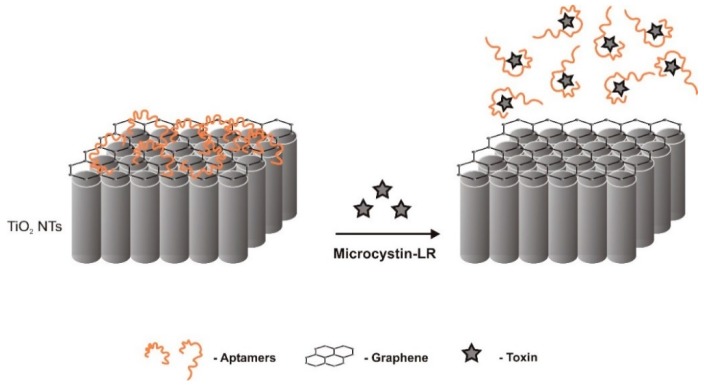
Label-free visible-light driven photoelectrochemical aptasensor for microcystin-LR (MC-LR) detection [52]. Aptamers are conjugated onto vertically aligned titanium dioxide nanotubes (TiO_2_ NTs) photoanode substrate, functionalized with graphene, where the DNA nucleobases are adsorbed via π-π staking. In the contact of MC-LR, aptamers leave graphene sheets and lead to the increased photocurrent.

A label-free visible-light driven photoelectrochemical (PEC) aptasensor was developed by Liu et al. [52] with high sensitivity and selectivity for MC-LR (Figure 8). PEC sensors are ultrasensitive because of the different forms of energy for excitation (light) and detection (current). Aptamers were attached through π-π stacking interactions between the graphene hexagonal cells and the DNA nucleobases. Functionalization of vertically-aligned titanium dioxide nanotubes (TiO_2_ NTs) by graphene enhanced the aptasensors visible-light response activity. This sensor combined favorable characteristics of both TiO_2_ and graphene to produce a very high sensitivity aptasensor with a LOD as low as 0.5 fM and the LRD of the photocurrent increment from 1.0 to 500 fM. Upon MC-LR binding, there is a dissociation of the aptamer from the surface of the electrode that leads to increased photocurrent. Selectivity was tested against contaminants that may interfere with MC-LR in field samples and it was proved to be very high. Another important characteristic of this MC-LR PEC aptasensor was its photocurrent stability. The RSD of consecutive readings, as the excitation light was turned on and off repeatedly, was only 0.3% (*n* = 6). The outstanding stability and repeatability are largely related to the steady loading of aptamers on the functionalized electrode surface and the potential confining effects provided by the special 3-D nanotubular structure of TiO_2_ NTs [52].

**Figure 9:**
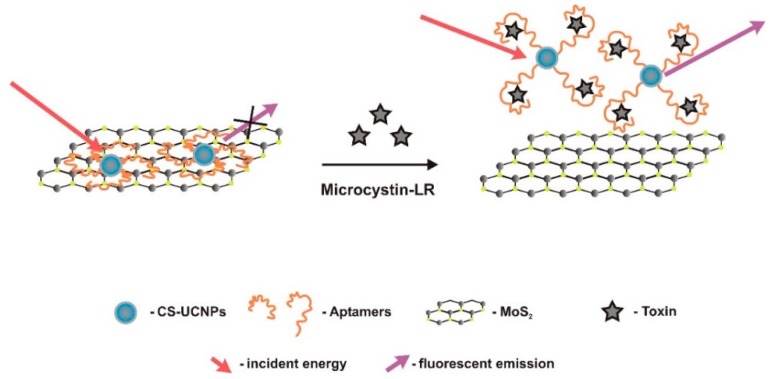
Optical aptasensor that uses the higher fluorescence of lanthanide ions doped core/shell upconversion nanoparticles (CS-UCNPs) and MoS_2_ nanosheets [53]. In the presence of microcystin-LR, aptamers combined preferentially with MC-LR, changing conformation, detaching from MoS_2_ and recovering fluorescence.

Lv et al. [53] build another ultrasensitive optical aptasensor, this time using the enhanced fluorescence of lanthanide ions doped core/shell upconversion nanoparticles (CS-UCNPs) and MoS_2_ (Figure 9). NaYF4 nanoparticles, co-doped with Yb and Tm, were first produced and used as seeds for the epitaxial growth of NaYF4: Yb shells to form NaYF4: Yb,Tm@NaYF4: Yb core/shell NPs. Two-dimension MoS_2_ nanosheets have a structure analogous to that of graphene with exceptional nanoelectronics, optoelectronics, and photovoltaics characteristics [84,94,95,96], showing high fluorescence quenching capacity and specific affinity to ssDNA [97], characteristics that are very convenient to biosensors. Polyacrylic acid functionalized CS-UCNPs were activated via EDC/sulfo-NHS chemistry, then avidin was added to form avidin-conjugated CS-UCNPs, and at continuation combined with biotin-aptamers. MoS_2_ adsorbed the aptamer-modified CS-UCNPs through van der Waals forces between the nucleobases and the basal plane of MoS_2_, causing energy transfer to the MoS_2_, and quenching of the fluorescence. After adding MC-LR, the aptamer conjugated preferentially with it, changing conformation, detaching from MoS_2_, and allowing for fluorescence recover. Upconversion fluorescence spectra of NaYF4: Yb,Tm@NaYF4: Yb core/shell NPs occurred at 980 nm, and MC-LR quantitative analysis was performed by measuring fluorescence response at 361 nm. LRD ranged between 0.01 μg/L and 50 μg/L, and the LOD was 0.002 μg/L.

**Figure 10:**
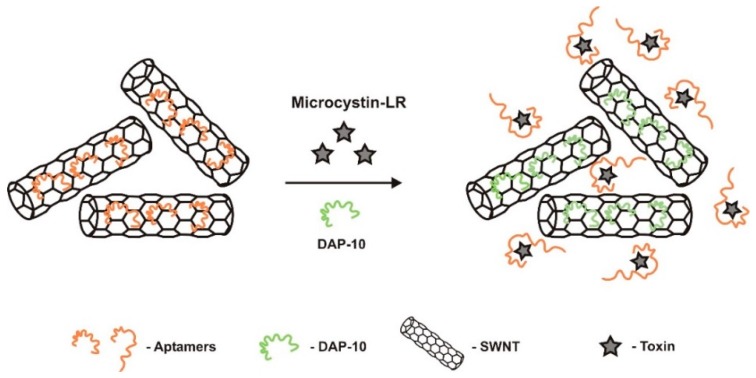
Optical aptasensor for ultrasensitive detection of microcystin-LR using single-walled carbon nanotubes (SWNTs), dapoxyl fluorescent dye, an aptamer with affinity to dapoxyl dye (DAP-10), and another aptamer with affinity to MC-LR, developed by Taghdisi et al. [35].

A novel ultrasensitive fluorescent aptasensor was developed by Taghdisi et al. [35] (Figure 10) for detection of MC-LR, using single-walled carbon nanotubes (SWNTs), dapoxyl dye and an aptamer (DAP-10) with affinity to dapoxyl dye. SWNTs were used to immobilize DAP-10 (5′-CAATTACGGGGGAGGGTGTGTGGTCTTGCTTGGTTCGTATTG-3′) and an unmodified MC-LR aptamer (Apt) that was used as a sensing ligand. Significantly different fluorescence intensity was measured in the presence and absence MC-LR. Without MC-LR, DAP-10 could not adsorb to the surface of SWNTs because it was loaded with Apt and no space was available. After adding dapoxyl to the supernatant and have the sample centrifuged, it becomes strongly fluorescent. When dapoxyl is attached to DAP-10 its fluorescence increases significantly because the surrounding gets less polar as compared to water. When the Apt binds to MC-LR it leaves free space on SWNTs surface for DAP-10 aptamer to attach, resulting in very weak fluorescence intensity of the sample after addition of dapoxyl. Apt was used without any modification, which increased the sensitivity of the sensor. The LOD was 138 pM (0.137 μg/L) and the LRD was from 0.4 to 1200 nM.

### 2.2. Marine Toxins

Marine biotoxins are classified into ten groups according to the chemical structure of the molecule, namely brevetoxin (BTX), okadaic acid (OA), palytoxin (PTX), saxitoxin (STX), cyclic imine (CI), domoic acid (DA), pectenotoxin (PTX), yessotoxin (YTX), azaspiracid (AZA), and ciguatoxin (CTX) [98]. Among these, the first four groups are the most relevant within the aptasensing field, along with some analogs, are described next.

#### 2.2.1. Brevetoxins

Brevetoxins are cyclic polyether lipophilic marine neurotoxins produced by some strains of dinoflagellates of the genera *Karenia* and *Chattonella.* The toxin is found initially inside the dinoflagellate cells but then exudates into the environment after the cells lyse or die [99]. BTX binds to Na^+^-channels in nerve and muscle cells, impairing normal voltage processes and causing an excessive influx of Na^+^ across membranes. Massive fish kills, poisoning of marine mammals and aquatic invertebrates have been reported. Consumption of these organisms by humans causes Neurotoxic Shellfish Poisoning (NSP). BTX-2 is the most common toxin responsible for NSP and only BTX has aptamers developed against it [37,66]. One aptasensor was developed upon one of these aptamers [37]. This toxin has a MW of 895.08 g/mol. It is heat and acid stable. 

**Figure 11:**
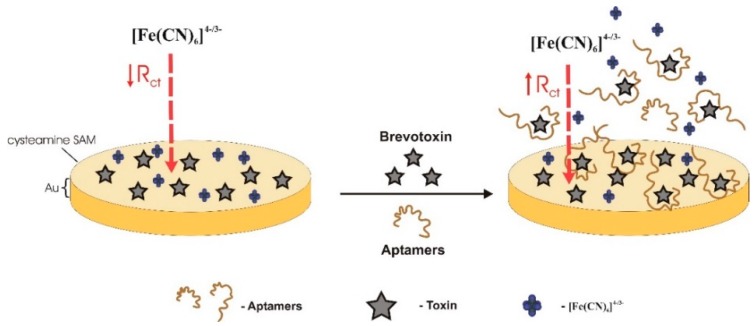
Label-free impedimetric competitive biosensor for brevetoxin-2 (BTX-2), developed by Eissa et al. [37], where competition is established between the BTX-2 immobilized on the self-assembled monolayer (SAM) of cysteamine on a gold electrode surface, and the free BTX-2 in solution, at a specific aptamer concentration. The change of charge transfer resistance (R_ct_) of the [Fe(CN)_6_]^4−/3−^ redox couple is the signal for electrochemical detection.

A label-free competitive impedimetric biosensor was constructed by Eissa et al. [37] to detect BTX-2. The toxin was immobilized through its hydroxyl group on the surface of the gold electrode, so as to keep exposing the same site of the molecule for DNA binding, as that when attached to the divinyl sulfone beads used for SELEX. The electrode gold surface was functionalized with a cysteamine self-assembled monolayer (SAM), and then, a bifunctional linker, 1,4-phenylene diisocyanate (PDIC), covalently linked the terminal amine groups of Cys/Au to the hydroxyl groups of the toxin. The competition was between the immobilized BTX-2 and the free BTX-2 in solution, at a given aptamer concentration (Figure 11). There was a change of resistance, of the [Fe(CN)_6_]^4−/3−^ redox couple, as the aptamers leave the sensor surface to bind to free BTX-2. The LOD of the sensor is 0.106 μg/L, the LRD is between 0.01 μg/L to 2000 μg/L, and K_d_ is 42 nM. Spiked shellfish extracts were analyzed using this aptasensor, showing non-significant interference from the matrix on the aptasensor response. In this study, the secondary structures of most of the obtained aptamers were typical stem-loop and hairpin-loop structures [66]. The aptamer selected with the highest affinity has a K_d_ of 4.83 μM, a LOD of 3.125 μg/L, and a LRD over the range of 3.125–200 μg/L. The EC and Codex Alimentarius Commission regulatory limit is 800 μg/kg shellfish meat. Considering that the aptamer is added after the presence of the toxin on the sensing area, this work does not really involve a true aptasensor.

#### 2.2.2. Okadaic Acid (OA)

Okadaic acid is a polyketide polyether lipophilic toxin, with a MW of 805 g/mol, derivative of a 38 carbon long fatty acid. It is produced by marine dinoflagellates of *Dynophysis* genus and has been isolated from black sponges (*Halichondria* sp.) and mussels (*Mytilus* sp.). More recently, a diversity of other invertebrate vectors has been described [100]. It causes diarrhea (Diarrhea Shellfish Poisoning—DSP), nausea, vomiting, and abdominal pain. OA is also a potent tumor promoter in rodent’s skin and glandular stomach, an inhibitor of protein phosphatases PP1 and PP2A, apoptosis inducer and ionophore.

A label-free electrochemical biosensor was developed also by Eissa et al. [50] for OA detection, based on a polycrystalline gold electrode, where a disulfide-modified aptamer was immobilized by Au-S interaction. The electrode surface was treated with MCH to reduce the nonspecific adsorption of the aptamers. The decrease of the resistance after OA binding was used as the sensor signal. The sensor showed a LOD < 0.070 μg/L, a LRD from 0.1 to 60 μg/L, and a K_d_ of 77 nM. The aptasensor did not cross-react with the similar structure toxins *Dynophysis*’ toxin-1 and 2 (DTX-1, DTX-2), nor with MR-LR or OA.

A dual fluorescence resonance energy transfer (FRET) aptasensor developed by Wu et al. [59] to target OA is described above in the MC-LR section since it is a duplex sensor. Its LOD for OA is 0.05 μg/L and the LRD is from 0.1 to 50 μg/L.

Another OA optical direct aptasensor was developed by Gu et al. [60], where aptamers were used to create a direct competitive enzyme-linked aptamer assay, an assay similar to ELISA in which the antibodies are substituted by aptamers [101]. A short biotinylated complementary sequence (5′-Biotin-GATGCGTCTCCCTCG-3′) to the aptamer competed with the free OA in the sample. OA aptamers are immobilized on the well plate, hybridized to the biotinylated complementary aptamer; the latter bound to avidin-catalase conjugate. Adding OA to the reaction system initiates competition with the complementary sequence for the OA aptamer. The higher the OA concentration, the fewer the complementary sequences attached to the OA aptamer. The complementary sequence in solution is then washed away, which decreases the amount of avidin-catalase available in the next step when H_2_O_2_ is added and leads to an increased amount of H_2_O_2_ that survived hydrolysis. Addition of gold trichloric acid resulted in different colors. Low levels of H_2_O_2_ reduce aggregation of gold trichloric acid to nanoparticles, which changed the color of the solution to blue. High levels of H_2_O_2_ formed non-aggregated gold nanoparticles, and the solution turns to red. Cross-reactivity was checked against DTX-1, DTX-2, domoic acid and SXT. The sensor showed a LOD of <0.010 μg/L, a wide LRD from 0.025 to 10 μg/L, a K_d_ of 40 ± 13 nM, good recovery rate (92.86–103.34%), and high repeatability (RSD of 2.28–4.53%).

**Figure 12:**
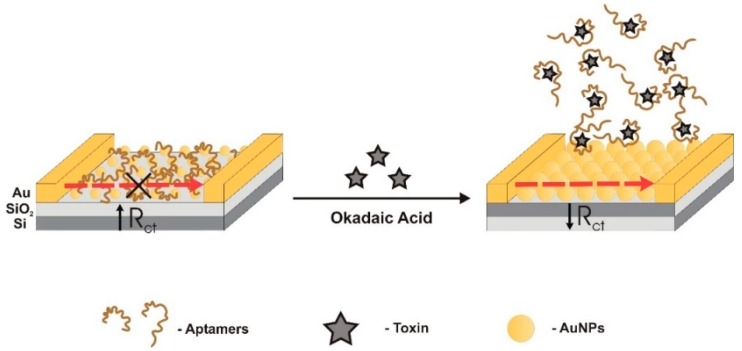
Electrochemical gap-based competitive assay, developed by Pan et al. [51], to detect okadaic acid, using gold nanoparticles (AuNPs). The self-catalytic growth of AuNPs creates conductive bridges. The presence of okadaic acid prevents the interaction of the aptamers and AuNPs, letting AuNPs sites exposed to the occurrence of catalytic growth, therefore inducing a large drop in charge transfer resistance (R_ct_).

Pan et al. [51] developed an electrochemical label-free gap-based competitive bioassay to detect OA. Interdigitated microelectrodes were modified with AuNPs and self-catalytic growth of AuNPs was used as conductive bridges (conductive tag) (Figure 12). Growth efficiency of AuNPs directly amplifies the signal and is related to the catalytic activity of AuNPs. When there are no OA molecules in the sample, aptamers adsorb electrostatically onto the AuNPs surfaces, fully blocking the AuNPs catalytically active sites and impairing AuNPs growth. When OA molecules are added to the sample, they bind to the aptamer preventing its adsorption to AuNPs, letting AuNPs sites exposed to the occurrence of catalytic growth, inducing a large drop in charge transfer resistance (R_ct_). In essence, AuNPs function in sensor gaps as conductive bridges. The aptamer used was developed by Eissa et al. [50]. The sensor showed a LOD of 1 μg/L, a LRD from 5 to 80 μg/L, and K_d_ was 77 nM. 

#### 2.2.3. Palytoxin (PTX)

Palytoxins are large (M_W_ = 2680.17 g/mol) and complex fatty alcohols, with a large polyhydroxylated partially unsaturated aliphatic backbone with eight double bonds. They have three nitrogen atoms, one on a primary amino group and the other two on two amide moieties. PTXs are produced by marine dinoflagellates (*Ostreopsis* sp.) and cyanobacteria (*Trichodesmium* sp.), and reported primarily in diverse anthozoan corals (*Palyhtoa* sp.). The toxic effects of PTX are related to dermal and ocular contact, as well as, inhalation exposure. PTX is neurotoxic and an intense vasoconstrictor, it causes death by muscle fatigue via opening the flux of Na^+^/K^+^ pumps and over stimulating the axons. 

**Figure 13:**
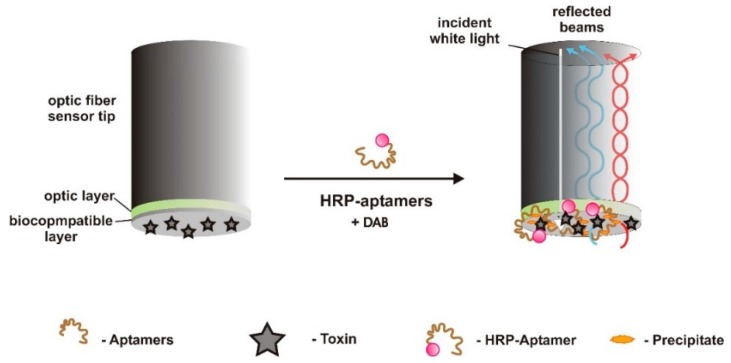
Real-time optical biosensor developed by Gao et al. [62] for palytoxin (PTX) detection, using coupled biolayer interferometry (BLI) and an enzyme-linked aptamer in a competitive binding assay. Binding events shift the interference spectrum of the reflected light. After being in contact with the sample, the biosensor tip with the PTX:HRP-aptamer complex attached was immersed in a 3,3′-diaminobenzidine (DAB) solution, resulting in the formation of a precipitate on the sensors surface, with subsequent signal amplification. HRP-horseradish peroxidase.

Gao et al. [62] designed a real-time optical biosensor for the detection of PTX using coupled biolayer interferometry (BLI) and an enzyme-linked aptamer. This sensor is very sensitive, based on a competitive binding assay, allowing for a very rapid quantification. BLI measures the interactions between biomolecules [102] by monitoring changes in the optical thickness at the surface layer of an optic fiber, where one of the molecules is immobilized, that occur upon biological binding events [102]. The biorecognition elements are aptamers labeled with horseradish peroxidase (HRP). Aptamers competitively bind either to PTX immobilized on the sensor’s surface, forming a monomolecular layer, or to free PTX on the sample. The binding events to the immobilized PTX cause a change in the interference spectrum of the reflected light, inversely proportional to the free PTX concentration in the sample, which is measured in real-time with precision and accuracy. This is an amine reactive 2nd generation biosensor, where PTX was bound to sensor’s surface by linking the terminal amine groups on PTX with the carboxylic groups on the biosensor surface, using EDC/NHS chemistry. After contact with the sample, the PTX:HRP-aptamer complex was immersed in a solution of 3,3′-diaminobenzidine (DAB), causing the formation of a polymeric product that precipitated directly on the biosensor surface, thereby generating a response of the BLI biosensor, and amplifying the sensor signal. The surface reflections are caused by the immobilized ligands layer on the surface, and the internal reference layer (Figure 13). The wavelength shift (Δλ) is related to the variation in the optical thickness of the sensor layer, generating a signal in the Octet system used (OctetRED96, ForteBio, Menlo Park, CA, USA). Octet Data Analysis Software (CFR Part 11 Version 6.x) was used to process the signal acquired. The biosensor showed a wide LRD of 200–700 ng/L, low LOD of 0.04 ng/L, high selectivity for PTX, good reproducibility and stability. The affinity of the aptamer selected against PTX was high (K_d_ = 0.843 nM).

#### 2.2.4. Saxitoxin (STX)

Saxitoxin (STX) is a light (M_W_ = 299.29 g/mol) carbamate alkaloid neurotoxin related to paralytic shellfish poisoning (PSP). STX analogs are grouped into four subgroups: carbamate, *N*-sulfo-carbamoyl, decarbamoyl, and hydroxylated saxitoxins [98]. More than 30 analogs have been described. STX interferes with voltage-gated sodium channels in neurons, and with calcium and potassium channels in cardiac cells, causing cellular malfunction and paralysis. STX is mainly produced by the marine dinoflagellates *Gymnodinium* sp., *Alexandrium* sp., *Pyrodinium* sp., *Protogonyaulax* sp., *Prorocentrum* sp. and *Coolia* sp.). In freshwater it is produced by several filamentous cyanobacteria species (*Anabaena* sp., *Aphanizomenon* sp., *Lyngbya* sp., *Cylindrospermopsis* sp., *Plantothrix* sp. and *Scytonema* sp.). Bivalve mollusks are the main vectors to humans, however, numerous other vectors such as crabs, lobster, and carnivorous snails have also been identified [100,103,104]. In the US, the regulatory action level is 80 μg STX equivalents/100 g tissue, similarly to EU where the regulatory limit is of 80 μg PSP per kg shellfish meat.

Handy et al. [67] in 2013, have selected the first aptamer with high affinity to a marine toxin, in the case a surface plasmon resonance sensor to detect STX. A hapten-carrier conjugate (keyhole limpet hemocyanin) was used for the first time on the SELEX method to select aptamers against small molecules, in this case, conjugated to STX. The conjugation transformed the small STX molecule into a larger (M_W_ = 350 kDa) protein moiety, that was linked to the surface of a solid support by surface chemistry (i.e., amine/epoxy reaction) to be used for coupling the conjugate. To eliminate aptamer candidate sequences that bound directly to the carrier, counter-selection was used. The estimated secondary structure for the STX aptamer selected featured four stem and loop segments, two of them involving the primers. In practice, this work does not fit into an aptasensor configuration.

Zheng et al. [68] have improved the aptamer selected by Handy et al. [67], making site-directed mutations and truncations, and creating an aptamer (M-30) with a 30-fold higher affinity. K_d_ for M-30 was 1.54 × 10^−3^ (s^−1^), whereas the K_d_ for the original Handy’s aptamer was 1.87 × 10^−2^ (s^−1^) and the association rate constant for M-30 was 1.20 × 10^4^ (M^−1^ s^−1^), one order of magnitude higher. Mutations were introduced to improve the conformation stability and to increase the interaction between the aptamer and the STX, bringing higher affinity. Increased structural stability was conferred by the formation of G-quadruplexes, which are characterized by extremely ordered architecture and stability [105]. Also, unnecessary nucleotides for STX binding were truncated, giving rise to the minimal functional core structure of M-30, reducing the synthesis cost. The *mfold* web server was used to predict which stem-loop structures of the aptamers were involved in the interaction with STX, and to determine their K_d_ values. Five truncated sequences of M-30 were synthesized according to predictions. The core nucleotides for a G-quadruplex were found to be positioned in the first and second stem-loop structures. The final optimized M-30 aptamer was synthesized without the third and fourth stem-loops, and the redundant nucleotides removed at both ends. The affinity of this minimal aptamer to STX was identical to that of the full-length M-30.

Hu et al. [106] have selected a very specific ssDNA aptamer that mimicked STX in antibody binding, with a dissociation constant (K_d_) in the nM range. Its high binding affinity and specificity were confirmed by indirect ELISA, indirect competitive ELISA, and equilibrium filtration method. The aim was to use this STX substitute, in the form of an aptamer, to replace the STX standard in a nontoxic detection assay, for STX detection in seafood products.

Alfaro et al. [54] have designed an original optical aptasensor that uses the next generation dye (Evagreen. Biotium, Inc., Fremont, CA, USA) for double-stranded DNA dying. This dye allowed to distinguish and measure the fluorescence signal from STX-binding aptamers at different STX concentrations. The folding configuration of the aptamer increased the amount of dsDNA area, as compared to the random one, allowing to determine a response pattern. However, the correlation between STX concentration and dsDNA fell deeply when quantification was performed in rough shellfish extracts. This was attributed to the quite low affinity (K_d_ = ~3.84 M) of the aptamer used, which was the one developed by Handy et al. [67]. Future trials need to use higher affinity aptamers to obtain a better performing aptamer-based method to use in field samples. This method was very rapid, requiring minute volumes of sample. Fluorescence was measured through high resolution melting analyses in a real-time thermocycler at 500/530 nm. Background noise is significantly diminished with Evagreen that uses a ‘release on demand’ mechanism, emitting fluorescence after changing configuration upon binding to dsDNA. A LRD from 15 to 3 mg/L and a LOD of 7.5 μg/L were attained.

#### 2.2.5. Gonyautoxin (GTX1/4)

Gonyautoxins are saxitoxin analogs. GTX1 and GTX4 are epimers (M_W_ 411.346 g/mol) featuring two guanidine groups and one sulfonic acid group. They commonly coexist as they convert into each other, in neutral or alkaline conditions, until reaching an equilibrium state [107]. GTXs are produced by various marine dinoflagellate species (*Alexandrium* sp., *Gonyaulax* sp., *Protogonyaulax* sp.), and found in bivalves, gastropods, and echinoderms [108].

A GTX1/4 aptamer was selected by Gao et al. [55] to build an optical aptasensor based on BLI. It is a label-free sensor that delivers results in real-time. When GTX1/4 molecules bound to or dissociate from the aptamers on the sensor surface, there is a change in the interference spectrum that results in varying biolayer thickness, which generated a response curve. A three-chip sensor was used, including a super streptavidin-coated (SSA), a streptavidin-coated (SA) and an amine reactive 2nd generation (AR2G) sensor. The response of the SSA aptasensor was significantly higher, mostly because more aptamers could be immobilized at the spatial structure of the sensor chip on the biolayer surface, resulting in a larger mass density variation. An advanced screening SELEX technology using graphene oxide (GO), was used with noteworthy advantages to developing aptamers for small molecules [109]. During the selection process, the ssDNA that was not able to bind to GTX1/4 was coupled on GO surface, via π-π stacking and hydrophobic interactions, while the ssDNA that could bind to the toxin remained in solution, bound to GTX1/4, and it was recovered by centrifugation and then amplified. Interference from the binding of aptamers from the pool to the conjugation side of the targets was circumvented, for the GTX1/4 was not immobilization during the selection process. This method selects more aptamers and aptamers with higher affinity than methods using magnetic beads. It is highly efficient and cost-effective as compared to traditional SELEX. Furthermore, the affinity of the GTX1/4 aptamer was increased when one of the stem-loops was truncated. Probably, after the truncation, binding sites on other steam-loops were exposed, so that the aptamer could better fold around GTX1/4, interacting further with it. The aptamer core sequence obtained had a higher affinity, with a K_d_ of 21.9 nM. The sensor LOD was 0.05 μg/L, the LRD ranged from 0.2 to 90 μg/L, it had good specificity, recovery rate (86.70–101.29%), and repeatability of 1.2%.

#### 2.2.6. Tetrodotoxin (TTX)

Tetrodotoxin is a non-proteic basic alkaloid neurotoxin (M_W_ = 319.27 g/mol) with a tricyclic structure and a positively charged guanidinium group. Although found mainly in the liver and ovaries of Tetraodontiform fish, TTX it is not synthesized by fish but produced by bacterial strains, of the Vibrionaceae and Pseudomonas families, which were isolated from the intestine of the puffer fish *Fugu vermicularis radiates* [110]. TTX has also been described in invertebrates such as octopus, crabs, and starfish [111], and in terrestrial vertebrates such as newts and tree frogs from North America. It blocks the site 1 of TTX-sensitive voltage-gated Na channels, blocking cellular communication on the nerve membranes surface, inhibiting the production and transmission of the action potential.

Fomo et al. [56] have developed the first electrochemical impedimetric sensor for tetrodotoxin. A GCEs were modified with a poly(4-styrenesulfonic acid)-doped polyaniline film (PANI/PSSA) by electrodeposition. A PANI/PSSA film was then glutaraldehyde-functionalized to immobilize amine-end functionalized TTX-binding aptamers by a covalent glutaraldehyde cross-linking. The resulting aptasensor (GC//PANI+/PSSA-glu-NH2-Apt) was then studied by cyclic voltammetry and electrochemical impedance spectroscopy. It was confirmed that the electrochemical properties of the sensor were modulated by the complexation of the analyte to the immobilized aptamer, mainly the R_ct_ of the PANI+/PSSA film, which was used as a reporter signal. Increased R_ct_ values were obtained against increasing TTX concentrations. The aptamer sequence was selected by Shao et al. [69]. Values of LRD, LOD, and sensitivity of the sensor were 0.23 to 1.07 μg/L, 0.199 μg/L, and 134.88 ± 11.42 Ω/μg/L, respectively.

**Figure 14:**
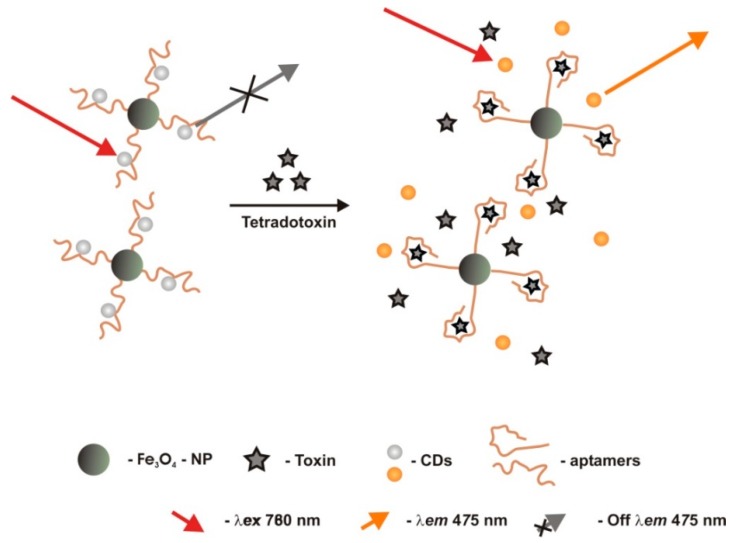
Optical aptasensor designed by Jin et al. [57], combining spectrofluorimetric methods, carbon dots (CDs), and upconversion fluorescence. Magnetic Fe_3_O_4_-nanoparticles (NPs) form Fe_3_O_4_/aptamer complexes, that then self-assembled with the CDs to form Fe_3_O_4_/aptamer/CDs nanocomposites, with upconversion fluorescence properties. When excited at 780 nm, UCF peaks at 475 nm; the fluorescence intensity increasing linearly with increasing tetrodotoxin log concentrations.

Another aptasensor for TTX was constructed by Jin et al. [57] combining spectrofluorimetric methods, carbon dots (CDs) and upconversion fluorescence (UCF) (Figure 14). Magnetic thiodiglycolic acid-stabilized Fe_3_O_4_ NPs were modified with the -NH_2_ terminated aptamer [56], to form a Fe_3_O_4_/aptamer complex, through carbodiimide-activated coupling. Then, self-assembling of CDs formed Fe_3_O_4_/aptamer/CDs nanocomposites, through π-π stacking, that exhibited simultaneously down-conversion fluorescence and UCF emissions. When excited at 780 nm, the UCF peaked at 475 nm upraising almost linearly with the Log [TTX] concentration, between 0.1 to 100 μg/L. LOD was 0.06 μg/L. The probe showed good sensitivity and high selectivity. With real biological samples, this biosensor showed high-performance analysis, with higher detection recoveries than other methods for TTX detection developed so far.

## 3. Discussion

The LODs of the aptasensors examined in the previous section have been decreasing from the nanomolar to the femtomolar order of magnitude in the past 20 years, due to the use of new nanomaterials, with outstanding characteristics and, new design strategies to produce and amplify the signal. All, except one aptasensor produced in the early 2000s, have LODs below the maximum concentration values set for the toxins that have concentration limits legislated. They are very selective and reproducible, and amenable to analyze water and biological tissues.

So far, only nine aquatic toxins have aptasensors developed for their detection. Twenty out of the thirty aptasensors analyzed here target freshwater toxins and only ten target marine toxins. Nearly half of the total of those aptasensors was designed to detect MC-LR. Despite the multiplex feasibility of aptasensors, only one was developed for more the detection of more than one toxin, MC-LR and OA, simultaneously.

All the aptasensors developed are DNA-based, except which uses RNA, and none is peptide-based. The little use of RNA aptamers is probably related to their limited stability, and to the much higher costs when compared to DNA-based aptamers. Not many strategies have been applied in developing aptasensors for aquatic toxins. Only electrochemical and optical transductors have been used up to now, mostly employing target induced structure switch (TISS) mode. Both types of aptasensors are adequate to detect aquatic toxins; however, electrochemical sensors can be further simplified, and in many cases, reduced to a very small chip connected to an electric circuit, and a drop of water or liquid that resulted from the extraction of the toxin from a biological matrix. On the other side, optical aptasensors may need a more complex microfluidic component to add the samples and reagents to cuvettes and, then at the end of the reading, to clean the cuvettes, apart from the electromagnetic wave radiation source and a detector. The exception is BLI-based optical aptasensors, where the probe may be immersed in the sample, and the results are produced in real-time, instead of in consecutive batches. 

In electrochemical aptasensors, aptamers may be immobilized in the sensing platform by chemical or physical bonds, while in optical sensors aptamers may also be in suspension, detached from any surface. When aptamers are detached or bound to surfaces by chemisorption, it is more difficult to recover them as they are in suspension and may be discharged with the samples analyzed. In this case, there is a continuous need for aptamers, as in the case of antibodies, which is expensive and not much environmentally friendly. In the laboratories, this may not be a problem, but in the case of continuous monitoring in sensors deployed, in oceanographic or hydrographic buoys, this may increase the costs related to increased number of visits to the buoys, for maintenance of the system. Surface chemistry techniques e.g. crosslinking, provide a vast array of tools for chemically joining the aptamers by a covalent bond to solid surfaces or particles e.g., electrodes, nanoparticles, well plates, membranes. Apart from crosslinking, bioconjugation also includes surface modification and labeling of biomolecules. The evolution of this area of chemistry increased enormously the possibilities of aptasensors design.

The possibility of taking a picture of the optical signal and analyze it by image analysis, with smartphone’s applications, or send it through the internet to be analyzed elsewhere, are good possibilities to reduce analysis time and speed up risk assessment and implementation of measures. Also, for final consumers, this kind of analysis may be feasible, simple and secure, to analyze water of food, if adequate kits are developed.

Up to now, antibodies are considered a standard and are preferred, on what biosensors are concerned. For a wider use of aptasensors, aptamer-based methodologies need to be considered, validated, and approved by national and international regulatory Agencies, related to public health, and food and water safety, as legal tools for aquatic toxins analysis. Aptasensors seem very adequate to detect emerging toxins, due to the velocity aptamers can be produced and to their portability. 

There are various advantages to the use of aptamers compared to antibodies on toxins detection. Aptamers are more amenable to detect small molecules and aquatic toxins are often small molecules. Also, regarding immunization, very potent toxins may kill the animals used to produce antibodies, while aptamers have no ethical concerns related to the use of animals, as they are produced in vitro. Aptamers synthesis is faster (weeks) and they have similar performance parameters to biosensors based on antibodies, featuring LOD values that may be well below the limits imposed by the legislation for aquatic toxins. Aptamers are more adequate than antibodies for field work and aquatic toxins monitoring, as they are much more chemically stable, robust at room temperature, their thermal denaturation is reversible, and minimal sample preparation is needed. Overall, aptamers are amenable for field studies, deploy in oceanographic buoys, store in the luggage of seafood and freshwater consumers, commercially shippable at ambient temperature, and have a long shelf life. Other practical aspects are their versatility related to target diversity, and modification with chemical groups form immobilization and labeling, allowing very diverse design and detection strategies. Multiplex aptasensors are easier to produce, based on the ability to using combinations of aptamers marked with different labels, that allow for multiple target detection, which is convenient in the case of aquatic toxins that may occur concomitantly with others. Binding to target molecules is reversible in the case of aptamers, allowing for multiple uses, as aptamers may be bound to the sensor platform and later be regenerated, and in many cases analyses are reagent-free. 

**Figure 15:**
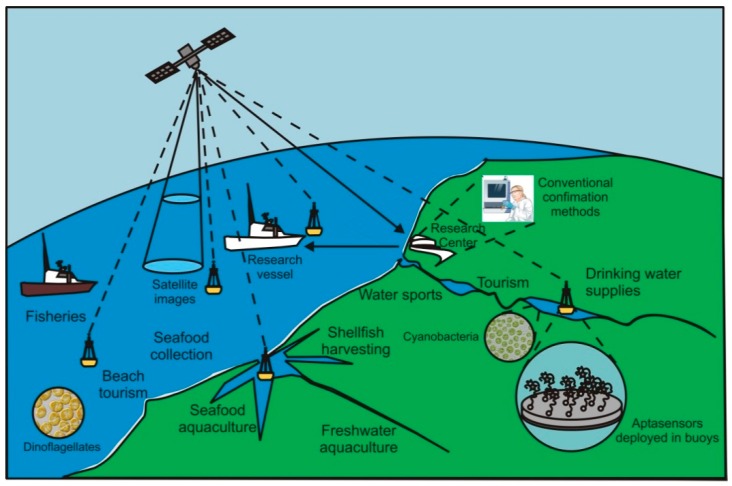
Integrated cyanotoxins monitoring scheme for fresh and marine waters, using buoys and a satellite. Hydrographic and oceanographic buoys emit data produced by aptasensors, and other physicochemical parameters measured by other sensors, to a satellite which forwards the information to a research center or specialized laboratory. When toxins are detected, water samples and sentinel organisms are collected by a research vessel and brought to the laboratory to follow standardized methods of analysis for each toxin. The satellite is equipped with remote sensing sensors for water masses surfaces image analysis, to produce data to correlate with those produced by ground sensors and laboratories, and feed models related to toxin-producing-microorganism blooms prediction.

Overall, the use of aptasensors for on-site toxins screening may turn out to be an advantageous approach, compared to conventional methods. On what concerns aquatic toxins, it is important that an analytical response is generated on-site, quickly and with low-cost, thereby allowing the implementation of safety or corrective measures (Figure 15).

The good analytical features of current aptasensors are most likely advising for their future commercialization. In general, it is important that moderate-to-high levels of toxins are always detected, which is a common feature among the several aptasensors. Yet, no alarm should be triggered by a positive response generated by a given aptasensor. This positive response could be real and lead to the implementation of the necessary measures, but it could also result from a cross-response of a parent compound. Thus, these devices could be observed mostly as effective screening tools, while a confirmative response is required by a conventional approach. 

Emerging toxins, that due to climate change and ballast waters, are increasingly being reported at more temperate waters, need to start to be monitored regularly in countries where there is evidence that they are becoming highly prevalent. Technical difficulties for the detection and quantification of some of these toxins must be overcome, and aptasensors may play a role on it in the future since sensitive, fast and reliable, detection and quantification methods are needed. Monitoring many toxins in many samples by the conventional methods, is a burden for State Agencies’ laboratories and private water-borne food producers, as these methods are labor intensive and expensive. Aptasensors function well even in complex matrices and are equally adequate to detect toxins directly in water as in extracted biological tissues with minimal sample processing, being adequate to use in sample screening stage with high throughput performance either using multiple sensors or multiplex sensors.

## Figures and Tables

**Table 1:** Description of the advantages of the use of aptasensors relative to sensors that use other recognition elements, and of the main characteristics, which are specifically suitable for aquatic toxins detection.

ADVANTAGES AND CHARACTERISTICS OF APTAMERS USE IN SENSORS	AMENABLE to DETECT SMALL MOLECULES, for aptamers are independent of ligand’s size or type, being convenient to detect small or large molecules or even whole cells. Animal immunization for low-molecular-weight targets without immunogenicity is not practical and aquatic toxins are often small molecules.
	AMENABLE to DETECT TOXINS, which may possibly kill live organisms or cells used to produce antibodies. Toxins do not have a toxic effect on aptamers.
	NO ETHICAL CONCERNS related to USE OF ANIMALS since aptamers synthesis is in vitro with no animals involved.
	PORTABILITY and ON-SITE REAL-TIME application are ideal features for risk assessment analysis and risk management of toxic blooms monitoring on water bodies used for drinking, bathing, food collection, and production.
	HIGH PERFORMANCE offered by aptamer-based sensors, similar to that offered by antibody-based ones, include selectivity, specificity, and accuracy. Their limits of detection may be well below the limits imposed by the legislation for aquatic toxins.
	STABILITY, in which DNA aptamers are very chemically stable; robust at room temperature; and their thermal denaturation is reversible. They are amenable for commercially shipping at ambient temperature and have a long shelf life. Due to its low stability, RNA aptamers are less suitable for environmental monitoring applications, unless stabilization techniques are used to protect them from nucleases. Overall, aptamers are amenable for field studies, deployment in oceanographic buoys or to be stored in the luggage of seafood and freshwater consumers.
	VERSATILITY, as they can be synthesized for a wide target diversity, modified by addition of chemical groups (e.g., −SH, −NH_2_, biotin) and labels (e.g., electrochemical probes, fluorophores, quenchers), adding or cutting nucleotides of the originally selected molecule, and being immobilized on surfaces quite easily, allowing diversity in designing, and detection strategies.
	TRAINED PERSONNEL or EXPENSIVE EQUIPMENT in specialized laboratories are not needed, which makes possible the use of sensors by water or food consumers or by low-tech remote laboratories.
	REVERSIBILITY of the binding between aptamers and target molecules, allowing multiple uses.
	EASY, HANDY and RAPID TO PRODUCE, as aptamers synthesis is very convenient, including the introduction of chemical modifications. They can be developed in weeks while antibodies need months.
	MULTIPLEXING ability by using combinations of aptamers marked with different labels in the same aptasensor, allowing for multiple target detection easily. Toxins may occur concomitantly with others.
	CONSISTENCY from batch-to-batch production, since aptamers are synthesized in vitro, thereby independent from the intrinsic variability of living organisms.
	NO PROPRIETARY in terms of aptamer sequence. Antibodies are often proprietary, while the sequence of aptamers is public to any researcher who wishes to make their synthesis and make use of them.
	LOW PRICE, aptamers cost is low when compared to antibodies. Once the nucleic acids sequence is known, chemical or enzymatic syntheses are possible at relatively low cost.
	MINIMAL SAMPLE PREPARATION is involved, even for complex matrices, due to their specificity.

**Table 2:** Type of sensors, architecture and, characteristics of the aptasensors and matrices analyzed, used in the detection of aquatic toxins.

Target Toxin	Type of Sensor	Design Strategy Mode *	Material Used as Transducer/Electrode/Platform/Redox Markers	Aptamer Immobilization Method	Linear Range of Detection (LRD)	Limit of Detection (LOD)	Reproducibility	Selectivity	Matrix Analyzed and Recover Rate **	References
ATX	Optic (label based)/fluorescence FRET	CR	fluorescent quantum dot nanocrystals and quencher fluorophore dyes	Aptamers were immobilized on streptavidin agarose beads and biotinylated at the 5′ end	Up to 100 μg/mL	-	-	-	-	[61]
	Electrochemical (label-free)/impedimetric	TISS	gold electrode/[Fe(CN)_6_]^4−/3−^ (redox marker)	Self-assembled monolayer (SAM) covalently bound through Au-S alkanethiol interaction	1–100 nM (0.165–16.5 g/L)	0.5 nM	6.5% (N = 7)	Good selectivity. CYN, MC-LR	spiked tap water and certified samples 94.8–108.6% (1.4–6.3%)	[31]
CYN	Electrochemical (label-free)/impedimetric	TISS	gold electrode/[Fe(CN)_6_]^4−/3−^	SAM covalently bound through Au-S alkanethiol interaction	0.1–80 nM (0.041–33.15 g/L)	0.1 nM (0.039 μg/L)	2.0–9.0% (N = 3)	Good selectivity. CYN, MC-LR, ATX-A	spiked tap water and certified samples 95.8–103.2% (2.0–9.6%)	[30]
	Electrochemical (label-free)/impedimetric	TISS	thionine–graphene nanocomposite modified glassy carbon electrode (GCE)/[Fe(CN)_6_]^4−/3−^	Covalently grafted through glutaraldehyde (cross-linked)	1–200 nM (0.39–78 g/L)	0.3 nM (0.117 μg/L)	1.2% (N = 3)	Good selectivity. OA, MC-LR	spiked lake water 96.3–104.6% (1.3–8.5%)	[33]
MC-LR	Optic (label-free)/Surface plasmon resonance	TISS	sensor chip (BIACORE international AB)	Affinity binding (biotin-streptavidin)	50.24–1005 M (50–1000 mg/L)	50.24 nM (50 mg/L)	10–20%	Poor selectivity. MC-YR, -RR	-	[48]
	Electrochemical (label-free)/square wave voltammetric	TISS	gold electrode/[Ru-(NH_3_)_6_]^3+^	SAM covalently bound through AU-S alkanethiol interaction	0.01–10 nM	0.0118 nM	-	Good selectivity. MC-YR, -LA	-	[28]
	No aptasensor was developed. The adsorption capacity of GO nanosheets was characterized.	TISS	graphene oxide (GO) nanosheets	carboxylic groups of GO are activated by 1-ethyl-3-(3-dimethylamino-propyl) carbodiimide hydrochloride/*N*-hydroy succinimideDC/NHS) to which NH_2_-aptamers covalently bind	0.5–1 ng/L	0.5 ng/L	-	Good selectivity. MC-RR, -LW, nodularin	spiked tap water 88.3%	[23]
	Electrochemical (label-free)/impedimetric	TISS	gold electrode/[Fe(CN)_6_]^4−/3−^	SAM covalently bound through AU-S alkanethiol interaction	0.05–100 nM	0.018 nM	3.52% (N = 5).	Good selectivity. against low concentrations of MC-RR	spiked lake, river and tap water 91.2–113.7% (2.6–4.2%)	[29]
	Electrochemical (label-free)/square wave voltammetric	TISS	graphene-modified screen-printed carbon electrodes (SPEs)/[Fe(CN)_6_]^4−/3−^	physisorption. π-π stacking interactions between the graphene hexagonal cells and the DNA nucleobases.	0.1–1000 nM	1.9 pM	-	Good selectivity. OA, MC-LA, -YR	spiked tap water and fish samples 91.7–98.1% (1.67–10.93%)	[36]
	Optic (label-based)/colorimetric	TID	polyethylene glycol (PEG) modified AuNPs	Aptamers hybridize to complementary DNA probes immobilized on the surface of AuNPs	0.1–250 nM	0.05 nM	3.6%	Good selectivity. MC-LA, -YR	river, lake, and pond water samples 89–100% and human serum 90–103%	[58]
	Optic (label-based)/fluorescence dual FRET	TID	green and red lanthanide upconversion nanoparticles (UCNPs) and quenchers	Affinity binding (biotin-streptavidin) to UCNPs	0.1–50 μg/L	0.025 μg/L	6.47% (N = 7)	Good selectivity. MC-LA, -YR	water, fish, and shrimps 99.1–110.8%	[59]
	Electrochemical (label-free)/Amperometric	TISS	graphene co-doped with BiOBr nanoflakes and nitrogen over an indium tin oxide (ITO) electrode	π-π staking interaction between graphene and aptamers	0.0001–100 nM	0.03 pM	3.46% (N = 5)	Good selectivity. MC-LA, -YR	spiked fish collected at local supermarket 97.8–101.6% (2.52–5.14%)	[32]
	Optic (label-free)/luminescence	TISS	graphene hydrogel co-doped with boron and nitrogen, self-assembled on a Ru(bpy)_3_^2+^ immobilization platform/GCE	electrostatic adsorption	0.0001–1 nM	0.03 pM	5.3% (N = 12)	Good selectivity. MC-LA, -YR	human serum, tap water and contaminated water 91.0−104.0%	[34]
	Electrochemical/impedimetric	TISS	cobalt (II) salicylaldimine metallodendrimer (SDD–Co(II)) doped with electro-synthesized silver NPs over a GCE	SAM covalently bound through Ag-S alkanethiol interaction	0.1–1.1 μg/L	0.04 μg/L	-	Good selectivity. Nodularin-R, MC-RR, -YR, 17-estradiol, zearalenone	spiked tap, distilled, and wastewater samples 94–115% (0.96–5.06%)	[38]
	Optic (label-based)/colorimetric	TISS	AuNPs	Physisorption at the AuNPs’ surface by coordination between the Au atoms and the N atoms of the exposed bases	0.5–7500 nM	0.37 nM	-	Good selectivity. acetamiprid, glyphosate, trichlorfon, clofentezine, atrazine	tap and pond water 95–102% (7.4–10.7%)	[49]
	Electrochemical (label-free)/photoelectrochemical	TISS	titanium dioxide nanotubes photo-anode substrate functionalized with graphene	Physisorption (noncovalent) π-π stacking interactions between the hexagonal cells of graphene and the DNA nucleobases.	1.0–500 fM	0.5 fM	0.3%	Good selectivity. monosultap, atrazine, glyphosate, omethoate, gramoxone, dipterex, and acetamiprid	-	[52]
	Optic (label-based)/fluorescent	TISS	lanthanide ions doped core/shell UCNPs (label) grafted on 2D MoS_2_ nanosheets (platform/quencher)	Affinity binding (biotin-streptavidin) to UCNPs. Van der Waals forces between nucleobases of UCNPs-aptamer and basal plane of MoS_2_ platform.	0.01–50 μg/L	0.002 μg/L	good	Good selectivity. MC-RR, MC-YR, MC-LA, OA	tap water and Tai lake water samples 94–112% (5.3–9.4%)	[53]
	Optic (label-based)/fluorescent	TISS	single-walled carbon nanotubes (SWNT-immobilizer) dapoxyl (fluorescent dye)	Aptamers interact with SWNTs through π-π stacking interactions	0.4–1200 nM	0.138 nM (0.137 μg/L)	-	Good selectivity. MC-LA, ochratoxin A, acetamiprid	water and serum samples 88.46–103.7% (2.6–8.4%)	[35]
MC-LR and MC-LA	Electrochemical/voltammetry	TISS	gold electrode/[Ru-(NH_3_)_6_]^3+^	Covalent self-assembly via thiol chemistry	0.01–10 nM	0.0075 (MC-LR) and 0.0106 nM (-LA)	-	Good selectivity (MC-YR)	-	[28]
MC-LR, MC-LA, MC-YR	Electrochemical/voltammetry	TISS	gold electrode/[Ru-(NH_3_)_6_]^3+^	Covalent self-assembly via thiol chemistry	0.01–10 nM	0.0077 (MC-LR); 0.0128 (-LA) and 0.0089 nM (-YR)	-	-	-	[28]
BTX-2	Electrochemical (label free)/impedimetric	CR	gold electrode functionalized with a cysteamine self-assembled monolayer (SAM); BTX-2 covalently attached through PDIC/[Fe(CN)_6_]^4−/3−^	-	0.01–2000 μg/L	0.106 μg/L	-	cross-selectivity to BTX-3 good selectivity to MC-LR and OA	spiked shellfish extracts 102–110% (3.4–6.0%)	[37]
OA	Electrochemical (label-free)/impedimetric	TISS	polycrystalline gold electrode/[Fe(CN)_6_]^4−/3−^	SAM covalently bound through AU-S alkyldisulfide interaction	0.1 to 60 μg/L	<0.070 μg/L	1.0–7.0% (N = 3)	Good selectivity. MC-LR, DTX-1, DTX-2	uncontaminated shellfish extracts 92%	[50]
	Optic (label-based)/fluorescence dual FRET	TID	green and red lanthanide UCNPs	Affinity binding (biotin-streptavidin) to UCNPs	0.1–50 μg/L	0.05 μg/L	6.24% (N = 7)	Good selectivity. DTX-1, DTX-2	water, fish, and shrimps 97.68–120.1%	[59]
	Optic (label-free)/colorimetric	TID/CR	biotinylated complimentary sequence to OA aptamer conjugated to avidin-catalase/gold trichloric acid and H_2_O_2_	Affinity binding (biotin-streptavidin) to well-plates	0.025–10 μg/L	0.01 μg/L	2.3–4.5%	Good selectivity. DTX-1, DTX-2, DA and SXT	spiked clam samples 92.86–103.34% (2.28–4.54%)	[60]
	Electrochemical (label-free)/impedimetric	TISS	AuNPs as conductive bridges	Electrostatic interaction	5–80 μg/L	1 μg/L	-	-	-	[51]
PTX	Optic (label-free) /biolayer interferometry	CR	PTX immobilized on optic fiber surface (transducer)	-	0.2–0.7 μg/L	40 pg/L	-	Good selectivity. OA, STX, MC-LR, BTX-A/B	water, clams, scallops, mussels 100.27–108.24% (2.27–6.76%)	[62]
STX	Optic /fluorimetric	TISS	Evagreen (Biotium, Inc) dye for double-stranded DNA/real-time thermocycler	-	15 μg/L–3 mg/L	7.5 μg/L	-	Good selectivity. GTX 2/3	rough shellfish extract	[54]
GTX1/4	Optic (label-free) /biolayer interferometry	TISS	GTX immobilized on optic fiber surface (transducer)	EDC/NHS to which NH_2_-aptamers covalently and affinity binding (biotin-streptavidin)	0.2–90 μg/L	0.05 μg/L	1.2%	GTX 2/3, neoSTX, STX	spiked shellfish samples 86.70–101.29%	[55]
TTX	Electrochemical/impedimetric	TISS	GCE modified with a poly(4-styrenesolfonic acid)-doped polyaniline film, where a TTX-binding aptamer was attached	grafted through a covalent glutaraldehyde cross-link	0.23–1.07 μg/L	0.199 μg/L	-	-	-	[56]
	Optic/fluorimetric	TISS	Fe_3_O_4_ nanoparticles modified with the aptamer self-assembled on carbon dots through π-π stacking	carbodiimide-activated covalently bound to Fe_3_O_4_ nanoparticles	0.1–100 μg/L	0.06 μg/L	-	Aflatoxin B1-B2, botulism neurotoxins A-B, *Staphylococcus aureus* enterotoxins A-B	human gastric juice, serum and urine 96.00–104.20% (1.44–4.21%)	[57]

* TISS—Target induced switch mode; TID—target induced displacement/dissociation; CR—Competitive replacement. ** Values in brackets correspond to relative standard deviation (RSD).

**Table 3:** Characteristics of the aptamers selected for the various aquatic phycotoxins and cyanotoxins, identifying modifications at 3’or 5′ ends to label or link the aptamers to the sensor platform and affinity to the target toxin.

Aptamer Sequence	Modification 1 (5′-End)	Modification 2 (3′-End)	Affinity (K_d_)	References
**Anatoxin-a (ATX-A)**				
5′-undisclosed-3′ ^1^	None	*QSY21−3′*	-	[61]
5′-TGG CGA CAA GAA GAC GTA CAA ACA CGC ACC AGG CCG GAG TGG AGT ATT CTG AGG TCG G-3′	5′−HO−(CH_2_)_6_−S−S−(CH_2_)_6_−disulfide-modified	None	27.14 ± 5.38 nM	[31]
**Cylindrospermopsin (CYN)**				
5′-GGC ATC AGG CAA CAA CCG ATG GTC CGG CCA CCC TAA CAA CCA GCC CAC CCA CCA CCC CGC CG-3′	5′−HO−(CH_2_)_6_−S−S−(CH_2_)_6_−disulfide-modified	None	20.25 ± 2.7 nM	[30]
5′-ATC AGG CAA CAA CCG ATG GTC CGG CCA CCC TAA CAA CCA GCC CAC CCA CCA CCC CGC CG-3′	5′−H_2_N-C_6_−Amine modified	None	-	[33]
**Microcystin-LR (MC-LR)**				
5′-undisclosed-3′	5′−biotin−	-	10^3^ M^−1^	[48]
5′-GGC GCC AAA CAG GAC CAC CAT GAC AAT TAC CCA TAC CAC CTC ATT ATG CCC CAT CTC CGC-3′	-	-	50 ± 12 nM	[32,34,35,36,49,52,58,59]
	5′−biotin−	NaYF_4_: Yb, Er/Mn UCNPs	50 ± 12 nM	[59]
	NaYF4: Yb, Tm@NaYF4: Yb core/shell UCNPs-avidin−5′−biotin−	-	50 ± 12 nM	[53]
	5′−SH(CH_2_)_6_−	-	50 ± 12 nM	[28,38]
5′-CCG GGG UAG GGA UGG GAG GUA UGG AGG GGU CCU UGU UUC C-3′ (RNA)	5′−H_2_N-C_6_−	-	-	[23,65]
5′-TTT TTG GGT CCC GGG GTA GGG ATG GGA GGT ATG GAG GGG TCC TTG TTT CCC TCT TG-3′	5′−SH(CH_2_)_6_−	-	-	[29]
**MC-LR and -LA**				
5′-CAC GCA CAG AAG ACA CCT ACA GGG CCA GAT CAC AAT CGG TTA GTG AAC TCG TAC GGC GCG-3′	5′−SH(CH_2_)_6_−	-	76 ± 13 nM (-LR) 106 ± 17 nM (-LA)	[28]
**MC-LR, LA and YR**				
5′-GGA CAA CAT AGG AAA AAG GCT CTG CTA CCG GAT CCC TGT TGT ATG GGC ATA TCT GTT GAT-3′	5′-SH(CH_2_)_6_−	-	705 ± 248 nM (-LR) 808 ± 123 nM (-LA) 193 ± 28 nM (-YR)	[28]
**Brevetoxin (BTX)**				
5′-GGC CAC CAA ACC ACA CCG TCG CAA CCG CGA GAA CCG AAG TAG TGA TCA TGT CCC TGC GTG-3′	-	-	42 nM	[37]
5′-undisclosed-3′	-	-	-	[66]
**Okadaic Acid (OA)**				
5′-GGT CAC CAA CAA CAG GGA GCG CTA CGC GAA GGG TCA ATG TGA CGT CAT GCG GAT GTG TGG-3′	5′-HO−(CH_2_)_6_−S−S−(CH_2_)_6_−	-	77 nM	[50,51]
	5′−biotin−	NaYF4: Yb, Ho UCNPs	50 ± 12 nM	[59]
5′-ATT TGA CCA TGT CGA GGG AGA CGC GCA GTC GCT ACC ACC T-3′	5′−biotin−	-	40 ± 13 nM	[60]
**Palytoxin (PTX)**				
5′-GGA GGT GGT GGG GAC TTT GCT TGT ACT GGG CGC CCG GTT GAA-3′	5′−biotin−	5′-HRP ^2^	0.843 nM	[62]
**Saxitoxin (STX)**				
5′-CCG TGG AAA CAT GTT CAT TGG GCG CAC TCC GCT TTC TGT A-3′	-	-	~3.84 M	[54,67]
5′-TTG AGG GTC GCA TCC CGT GGA AAC AGG TTC ATT G-3′	-	-	133 nM	[68]
**Gonyautoxin (GTX)**				
5′-AAC CTT TGG TCG GGC AAG GTA GGT T-3′	5′-Biotin 5′−H_2_N-C_6_−	-	21.9 nM	[55]
**Tetrodotoxin (TTX)**				
5′-AAAAATTTCACACGGGTGCCTCGGCTGTCC-3′	5′-NH_2_-	-	-	[56,57,69]

^1^ Despite the aptasensor architecture described being for anatoxin-A, the aptamer used was selected against thyroxin (T4), a similar size molecule used as surrogate due to the high toxicity of ATX-A. ^2^ HRP—Horseradish peroxidase.
